# The rational design, biofunctionalization and biological properties of orthopedic porous titanium implants: a review

**DOI:** 10.3389/fbioe.2025.1548675

**Published:** 2025-02-26

**Authors:** Chunliang Guo, Tao Ding, Yuan Cheng, Jianqing Zheng, Xiule Fang, Zhiyun Feng

**Affiliations:** ^1^ Wuxi People's Hospital, Wuxi, Jiangsu, China; ^2^ Nanjing Medical University, Nanjing, Jiangsu, China; ^3^ Wuxi Xishan NJU Institute of Applied Biotechnology, Wuxi, Jiangsu, China

**Keywords:** additive manufacturing, 3D printing, orthopedic implants, surface modification, environment-responsive, bone tissue engineering

## Abstract

Porous titanium implants are becoming an important tool in orthopedic clinical applications. This review provides a comprehensive survey of recent advances in porous titanium implants for orthopedic use. First, the review briefly describes the characteristics of bone and the design requirements of orthopedic implants. Subsequently, the pore size and structural design of porous titanium alloy materials are presented, then we introduce the application of porous titanium alloy implants in orthopedic clinical practice, including spine surgery, joint surgery, and the treatment of bone tumors. Following that, we describe the surface modifications applied to porous titanium implants to obtain better biological functions. Finally, we discuss incorporating environmental responsive mechanisms into porous titanium alloy materials to achieve additional functionalities.

## 1 Introduction

In the 21st century, human beings are entering the aging society at an accelerated pace (Rudnicka et al., 2020), therefore the demand and requirements for biomedical materials based implant devices are increasing. Bone is the main support structure of the human body, but it is also susceptible to injury and disease, such as infection, trauma, and tumors (Wang et al., 2024a). Traditional orthopedic treatments, such as internal fracture fixation, have certain limitations, for instance, traditional steel plates cannot meet the needs of repairing bone defects or promoting bone tissue regeneration (Islam et al., 2025). Autologous bone has long been considered as the best alternative material for tackling bone defects, but factors such as the limited amount of bone available from the autologous body, the new trauma that can be caused by the bone extraction process, and the poor quality of pathologic bone limit the extensive use of autologous bone grafting (Li et al., 2018).

Thus, the need for effective, sustainable and personalized methods of bone tissue repair has driven the development of biomaterials science and technology. To meet the urgent clinical needs, bone tissue engineering has emerged. A variety of bone replacement materials with good mechanical properties and bionic structure have been developed, achieving satisfactory clinical therapeutic outcomes (Mochi et al., 2022). Titanium and its alloys are widely used in clinical trials and research due to their mechanical performance, corrosion resistance and non-magnetic properties (Pobloth et al., 2018). However, the elastic modulus of titanium alloy implants currently used in clinical applications reaches 90–115 GPa, which is much higher than that of human cortical bone and cancellous bone or cartilage (Pan et al., 2021a), the huge gap of elastic modulus causes an imbalance in the distribution of stress between the implant and the surrounding bone tissues, leading to stress shielding, which induces the loosening of the implant with respect to the bone tissues (Raffa et al., 2021).

It has been found that some porous materials have an elastic modulus that matches human bone tissue, which is an effective means to solve the elastic mismatch between implants and human bone (Maksoud et al., 2022), at the same time, the large number of pores that exist within the porous material is more conducive to the growth of tissues such as osteoblasts and blood vessels, which significantly promotes osteogenesis (Bose et al., 2018). Thus, porous materials are regarded as ideal materials for orthopedic implants (Losic, 2021), which have drawn extensive attention from both the academic and industrial field.

In this review, we discuss the development and current status of orthopedic porous titanium alloy implant technology, and focus on the development of porous titanium alloys through additive manufacturing technology, porous structure design, surface modification technology, microenvironment responsive and modulation, and current clinical applications to further address the major difficulties in orthopedic implant design, including stress caused by mismatch of elastic modulus between the implant and human bone tissue shieldingissues, bone regeneration obstacles caused by biologically inert surfaces, and biofilm-related periprosthetic infections. Meanwhile, the future development trend of orthopedic porous titanium implants is also prospected in the end of this review.

## 2 Bone characterization and orthopedic implant design requirements

### 2.1 Skeletal structure and properties

The composition of human bone varies with age, anatomical location and nutritional status. In general, bone mineral makes up about 50%–70% weight of adult human bone, organic matrix takes another 20%–40%, leaving about 5%–10% for water, and about 1%–5% for lipids (Gelse et al., 2003). About 90% of the organic matrix of bone consists of type I collagen, a triple helix molecule composed of two identical α-1 chains and a single α-2 chain (Avioli, 1991). Bone minerals make up about 75% of bone tissue and most of the bone minerals are in the form of hydroxyapatite, which consists of microcrystals with other components including carbonates and magnesium, providing the rigidity and strength for the bones to bear weight (Zhang et al., 2017a).

Bone can be divided into two categories based on structure and density: trabecular bone (cancellous bone) and cortical bone (dense bone). Cancellous bone is located in the interior of the bone and is spongy, consisting of interwoven trabeculae arranged in a 50%–90% porosity, which grow in the direction of extended stress to bear greater weight (Rho et al., 1993), while cortical bone is dense and distributed on the peripheral surface of the bone and consists of tightly arranged bone plates with a porosity of 3%–5% (Kenny and Raisz, 2002).

Bone is a metabolically active organ that undergoes constant remodeling throughout its life, and the remodeling cycle involves interactions between osteoblast and osteoclast and is regulated by human hormone levels and local factors (Hadjidakis and Androulakis, 2006). Bone remodeling consists of three successive phases: resorption, reversal and formation, where osteoclasts remove mineralized bone, followed by osteoblasts forming the bone matrix. Bone remodeling helps to adjust the bone structure to meet changing mechanical demands and responsible for repairing defects and preventing the accumulation of old bone (He and Li, 2018).

### 2.2 Orthopedic implant design requirements

Ideal orthopedic implants should be similar to natural bone in the aspect of structure and performance, providing both physical function and bioactivity that promotes the regeneration of bone tissue under injury. Ideal orthopedic implants should have several properties: (i) biocompatibility; (ii) biodegradability; (iii) osteoconductivity and osteoinduction; (iv) plasticity and strength; (v) microenvironment on the surface of the material (Niinomi, 2003). Titanium and its alloys meet the key criteria required for the design of orthopedic implants and have become the most widely used metallic materials in the manufacture of existing implants. However, titanium is biologically inert and requires geometry optimization and surface modification to achieve desired clinical performance (Liang et al., 2019). In addition, since bone has microstructures that exhibit spatial anisotropy, ideal implants should have similar hierarchical structures in multiple dimensions to fulfill the mechanisms required for bone growth and to facilitate osseointegration induction (Velásquez-García and Kornbluth, 2021).

With advances in medical technology, patients are no longer satisfied with implants merely for pain relief and restoration of basic motion, but also for long-lasting and maximum restoration of their original function. This requires a new generation of implants with a better anatomical and kinematic match to individuals. Clinical existing limb fixation plates, artificial joints, spinal titanium cages and fusion devices, etc., are all of uniform design, assembly-line production, whose design and manufacturing mode is conducive to the formation of standardized treatment plans, which adapted to the majority of patients, but there are still a lot of patients encountered with mismatch of implant size or geometric configuration (Perera et al., 2021), which increases the risk of surgery and affects the surgical results.

Personalized medicine is promising and received much attention in the 21st century (Potyondy et al., 2021). Additive Manufacturing is an important branch of Rapid Prototyping Technology, which integrates Computer-Aided Design, Computer Numerical Control laser-assisted modeling technology and material technology (Van Bael et al., 2012), and is able to accurately design the physical parameters such as pore size, porosity, pore shape and surface morphology of the implant material, which pocessesbetter characters than traditional orthopaedic implants, can not only produce personalized implants that are more desirable in terms of biocompatibility and mechanical properties, but also make it possible for personalized and customizable implants.

## 3 Application of porous titanium alloys in orthopedic implants

### 3.1 Additive manufacturing technologies

Traditional implants are usually manufactured through molds or material reduction processes, and the production cost of single pieces or small quantities of complex products is often expansible. Meanwhile, traditional manufacturing methods can only mimic the biological appearance and process a fully dense or fully porous structure.

In contemporary manufacturing, a diverse array of techniques are employed to fabricate materials with specific pore structures. For instance, fibre or mesh sintering can produce materials with high porosity and large specific surface areas, which are widely utilized in filtration and burner energy applications (Li and Zhou, 2022). Space Holder Sintering is capable of manufacturing porous materials with intricate pore distributions (Adamek et al., 2022), while dynamic freeze casting enables the production of materials with directional pore structures (Christiansen et al., 2020). In contrast, additive manufacturing technology stands out as it can create highly complex and customized structures, particularly geometries that are challenging to achieve using conventional methods. This versatility extends to a broader range of materials and application scenarios.

Metal additive manufacturing technology has been developed for more than 20 years (Cai, 2015), benefiting from the development of 3D printing technologies and equipment such as electron beam melting and selective laser melting, according to the mechanical, chemical, and biological properties, with high precision and efficiency. It is possible to complete the manufacturing of small parts according to the mechanical, chemical and biological properties, what is more, the microporous structure can be controlled to realize the perfect reproduction of the real bone tissue (Papaefstathiou et al., 2022).

The process of additive manufacturing of porous titanium implants can be divided into four steps: (i) acquiring patient imaging data such as CT and MRI, (ii) creating a digital model of the implant based on the patient’s condition through CAD software, converting it into a series of 2D layer slices and saving it as STL data, (iii) computer-controlled, layer-by-layer melting of the metal material to print and shape it, (iv) further imparting biological functions through grinding, coating and surface modification techniques to further critical biological functions (Zhen et al., 2019) Figures 1, 2.

**FIGURE 1:**
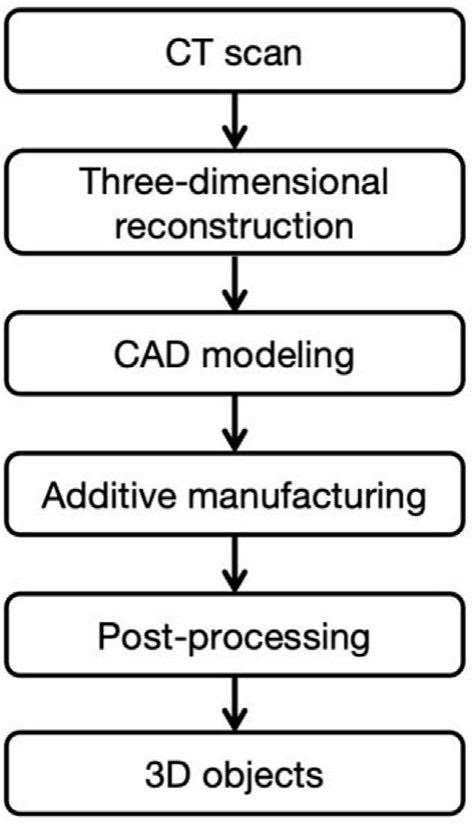
Flowchart of additive manufacturing.

**FIGURE 2:**
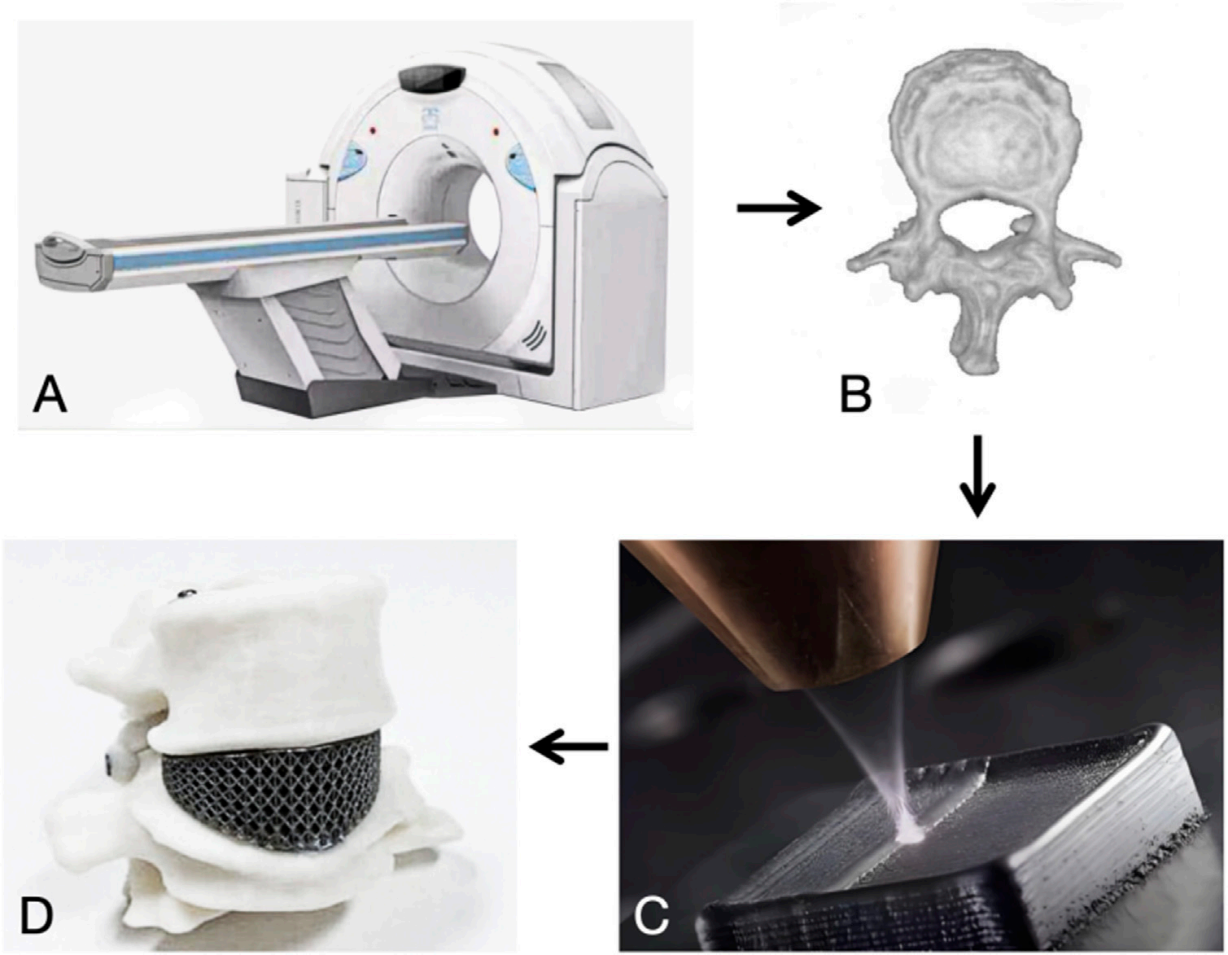
**(A)** CT scan. **(B)** Three-dimensional reconstruction. **(C)** Additive manufacturing. **(D)** 3D objects.

Traditional machining processes such as casting or subtractive manufacturing require customized molds or cutting of raw materials, and the production cost is usually high for single or small quantities. In contrast, additive manufacturing technology can be completed from design to manufacturing a product within 24 h, which greatly shortens the delivery time (Li et al., 2021a). At the same time, additive manufacturing technology can be personalized according to the morphological characteristics of the patient’s native bone, without being restricted by the differences in the patient’s anatomical structure, to create an implant that perfectly matches the patient, thus reducing the damages and improve the prognosis (Thomsen et al., 2005).

The layer of titanium powder melted by 3D technology is about 150 μm of thickness, which is close to the size of bone trabeculae of 100–140 μm (Kou and Tan, 2010). The bone trabeculae grow in the direction of delayed stress, which is not a homogeneous pore structure, so the non-uniform void structure can better mimic the biological structure of bone, and historically, the majority of non-uniform pore structures came from the method of reverse engineering the patient’s CT after three-dimensional reconstruction (Zhang et al., 2017b). With the proposal of VoronoiTessellation mathematical modeling method in recent years, it is able to construct an approximate model of biomimetic heterogeneous porous materials (MacBarb et al., 2017), and the controllable pore and surface morphology design is more conducive to the adhesion and proliferation of osteoclasts to achieve a strong biofixation between the implant and the bone tissues and to promote bone healing (El-Hajje et al., 2014). At the same time, by mimicking the natural structure of cancellous and cortical bone, the pore size and porosity of the material can be adjusted to obtain the appropriate modulus of elasticity and strength, so that the shape and mechanical properties of the implant can achieve a dual fit with the natural bone (Weber, 2019).

### 3.2 Materials used in additive manufacturing of orthopedic implants

The selection of materials for additively manufactured orthopaedic implants is crucial, as they must possess good biocompatibility, mechanical properties and corrosion resistance.

Stainless steel was one of the earliest metal materials used in orthopaedic implants, known for its corrosion resistance and strength (Marcomini et al., 2014). It is cost-effective to produce and can be shaped into various forms, with high strength and toughness, making it suitable for fracture fixation devices and artificial joints (Uhthoff et al., 2006). However, stainless steel corrodes in physiological environments, releasing metal ions that may be toxic to the human body, particularly nickel ions. The release of nickel ions can cause allergic reactions, tissue inflammation and osteolysis. Moreover, corrosion diminishes the mechanical properties of implants, increasing the risk of fracture (Agarwal et al., 2025). Cobalt alloys are primarily used in arthroplasty devices, exhibiting high mechanical strength, good wear resistance and corrosion resistance in body fluid environments (Sun et al., 2024). Although cobalt alloys are biocompatible, they are less biologically active. Their surface is typically biologically inert, resulting in weak bonding with bone tissue and potential implant loosening and failure. Additionally, the surface properties of cobalt alloys may impair cell adhesion and growth, thereby hindering the bone healing process (Safari-Gezaz et al., 2025). Titanium alloys are now of great interest due to their excellent biocompatibility and mechanical properties. The new β-type titanium alloys, enriched with elements such as Nb, Zr and Mo, have a significantly reduced modulus of elasticity, closely resembling human bone tissue while maintaining high strength and biocompatibility, thus being widely used in orthopaedic implants. The surface of titanium alloys usually forms a stable and firmly bonded oxide film, which is the primary reason for their corrosion resistance (Feng et al., 2024). Furthermore, surface modification enhances the corrosion resistance of titanium alloys by preventing chloride ions and proteins in body fluids from corroding the oxide film, which could otherwise lead to dissolution and stripping.

Polymer materials are also extensively used in orthopaedic implants, particularly polyether ether ketone (PEEK) and polylactic acid (PLA). PEEK overcomes the drawbacks of metallic materials, such as stress masking and radiation artefacts, and enables the rapid manufacturing of customised PEEK bone implants via additive manufacturing techniques (Li et al., 2015). PLA, a biodegradable polymer widely used in bone tissue engineering, can gradually degrade *in vivo* to promote natural bone tissue regeneration (Tajbakhsh and Hajiali, 2017). However, its relatively weak mechanical properties limit its use in load-bearing sites.

Composites integrate the advantages of metals and polymers to achieve better biocompatibility and mechanical properties. Metal-polymer composites enhance these properties by combining metal particles or fibres with a polymer matrix. For instance, titanium-PEEK composites have demonstrated excellent performance in orthopaedic implants for complex clinical needs (Tsai et al., 2021). Ceramic-polymer composites combine the high hardness of ceramics with the flexibility of polymers to provide good biocompatibility and mechanical properties. Hydroxyapatite-PLA composites, for example, have shown excellent osteoinductive ability and biocompatibility in bone tissue engineering (Fan et al., 2022a).

### 3.3 Pore size and structural design of porous titanium implants

The structural features of additively manufactured porous titanium alloy materials include pore size and void ratio, pore shape and surface morphology (Van Bael et al., 2012), in addition to the direct impact on mechanical properties, the porous structure provides important biological properties of the implant. A suitable pore structure facilitates osteoblast growth and migration and proliferation of osteogenic sites, allows nutrient and oxygen transfer through the structure, and supports vascular infiltration and growth into the structure, all of which can promote bone growth within the implant (Hollister, 2005).

#### 3.3.1 Uniform and gradient aperture design

Gradient pore size materials are a class of porous materials with gradual changes in pore size, porosity and solid composition phase. Bone trabeculae grow in the direction of extended stress and are not homogeneous pore structures, so non-uniform pore structures can better mimic the biological structure of bone and improve biocompatibility. By adjusting the size and relative density of pores, it not only endows open-pore structure high strength while possessing high porosity, but also optimizes the growth of bone tissue and adapt to changing stresses in specific regions (Faverani et al., 2014).

Most of the existing implants in orthopedic clinics are ordinary titanium alloy products, and the modulus of elasticity of solid titanium alloy implants is much higher than that of human cortical bone, cancellous bone or cartilage (Niinomi and Nakai, 2011). Since the modulus of elasticity of titanium alloy is far beyond that of the surrounding bone tissue, the force received cannot be normally transmitted to the surrounding, resulting in stress shielding phenomenon (Wang et al., 2023a), which causes atrophy or even necrosis of the bone tissue nearby, resulting in loosening or dislodging of the implant, and ultimately leading to shortening of implant life. Porous design can effectively reduce its elastic modulus and better match the surrounding bone tissue. The gradient pore size design can further optimize the stress distribution in a uniform way.

**FIGURE 3:**
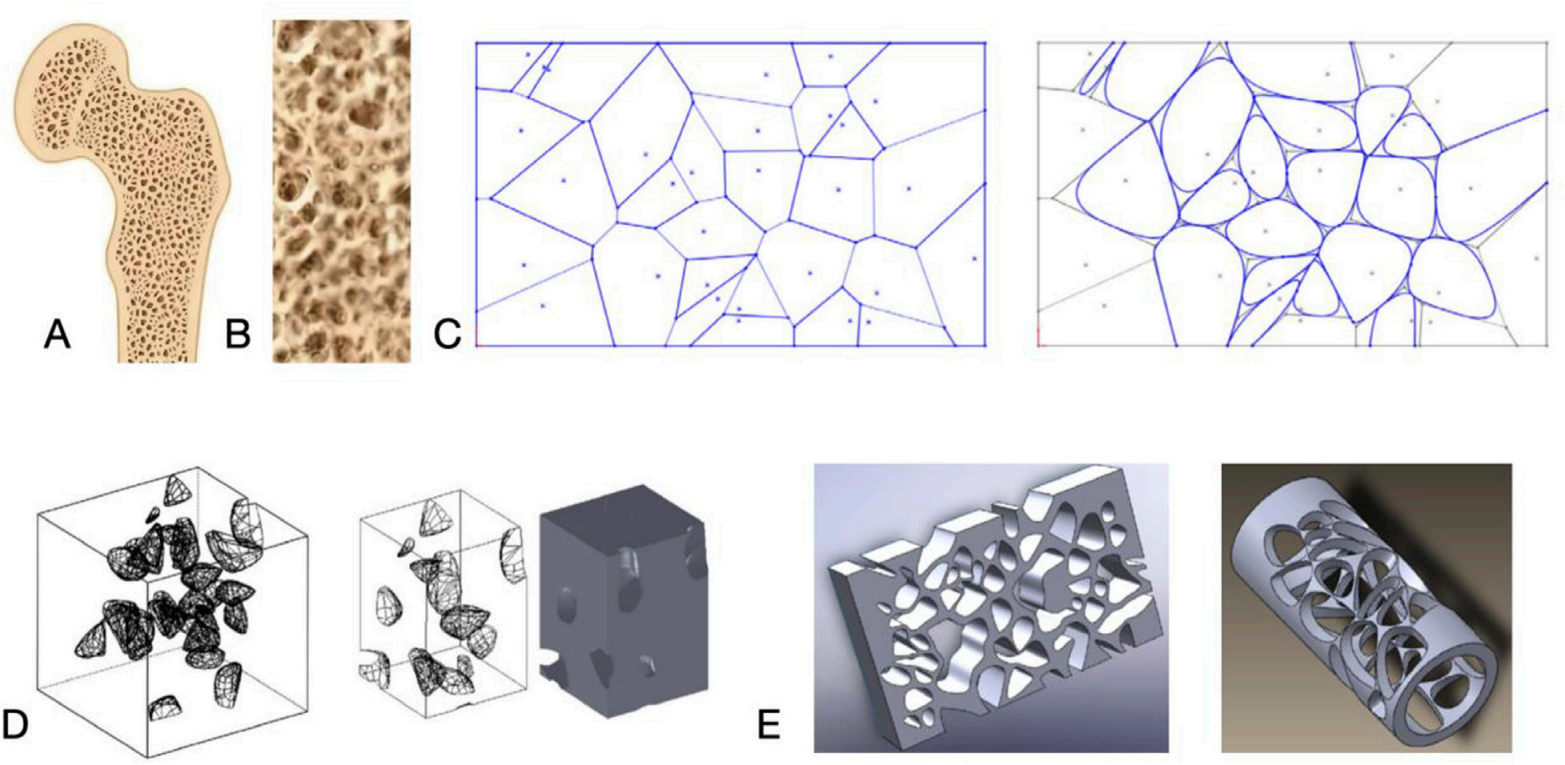
**(A)** Cross-section of bone. **(B)** Trabecular bone morphology. **(C)** Nature inspired geometric representation for irregular porous structure modeling. **(D, E)** 3D porous structures. Reproduced with permission (Kou and Tan, 2010).

Over small of the pore size in implant materials will hinder bone tissue growth in, which should be adjusted into around 600 μm has a good ability to induce new bone formation, while with the increase of pore size, when the pore size reaches 600 μm, the mechanical properties of the material, including the compressive strength, will be significantly reduced (Liu et al., 2017). Therefore, not only the elastic modulus is reduced by porous material to reduce the stress shielding phenomenon, but also to make the stress distribution more uniform, the porous implant is designed through a gradient to transmit a greater force by a structure with a small pore size and high stiffness to match the cortical bone, and a structure with a large pore size and low stiffness to match the cancellous bone, respectively, to induce the bone tissues to grow in to promote the osseointegration Figure 3.

Orthopedic implants have high requirements for fatigue resistance to ensure the safety, stability, and long-term performance of the implants. Studies have shown that fatigue strength is highly correlated with porosity, and the fatigue strength decreases with the increase of porosity Zhao et al. (2018). Itälä et al. (2001) found that, under equal strain conditions, mechanical properties of materials depend on their structure and gradient, and fatigue cracks in the cyclic deformation process of the components in the sequential sprouting, resulting in the continuous redistribution of the stress in its various layers, which significantly inhibits fatigue cracks in the porous material expansion, and effectively improves the fatigue resistance of the implant.

#### 3.3.2 Pore design of porous titanium based materials

Pore design has a significant impact on the biomechanical properties of plant entry as well as the osteointegration capacity. Existing studies have shown that porous materials with pore sizes less than 50 μm limit osteoblast growth (Yue et al., 2014), and various pore shapes (e.g., cuboidal or rhombic) were successful in inducing inward growth of bone tissues in the range of 100–600 μm Taniguchi et al. (2016). Ran et al. (2018) found that a pore size of 632 μm had stronger osseointegration fixation than a pore size of 309 vs. 956 μm in a study that observed the implantation of a titanium plate in the tibia of rabbits, while a pore size of 632 μm had the strongest bone growth at weeks 2 and 4, respectively. The results of *in vitro* and *in vivo* evaluations of different pore sizes and fabrication methods confirmed that smaller pore sizes (<100 μm) impeded the entry of cells, nutrients, and transport of oxygen inward, leading to poor vascularization and bone regeneration (Biemond et al., 2011). Larger pores (>900 μm) decrease the inward bone growth ratio, which may be associated with a reduction in the rate of local bone regeneration and effective contact between bone tissue and the implant (Biemond et al., 2013).

In addition to the pore size and porosity of the material, the pore geometry and surface morphology are also important for osteoinduction. Van Bael et al. (2012) concluded from *in vitro* cellular experiments that right-angle pore shapes are more favorable for cell growth, and that obtuse angles are more likely to result in cellular buildup and clogging than acute angles.

Meanwhile, pore size and porosity directly affect the mechanical properties of implants. With the increase of pore size and porosity, the elastic modulus and yield strength of porous titanium alloy materials gradually decrease (Fujii et al., 2022). Li et al. (2023a) prepared a serial of porous titanium alloys with different pore sizes and porosities, and analyzed the effect of the porosity on the compression properties through tests and simulations. Liu et al. (2022a) designed a multistage gradient porous titanium implant, and the structural performance of which was found to be the best through finite-element simulation analysis, it was found that the implant with a porosity of 59.86% had the best structural performance.

**FIGURE 4:**
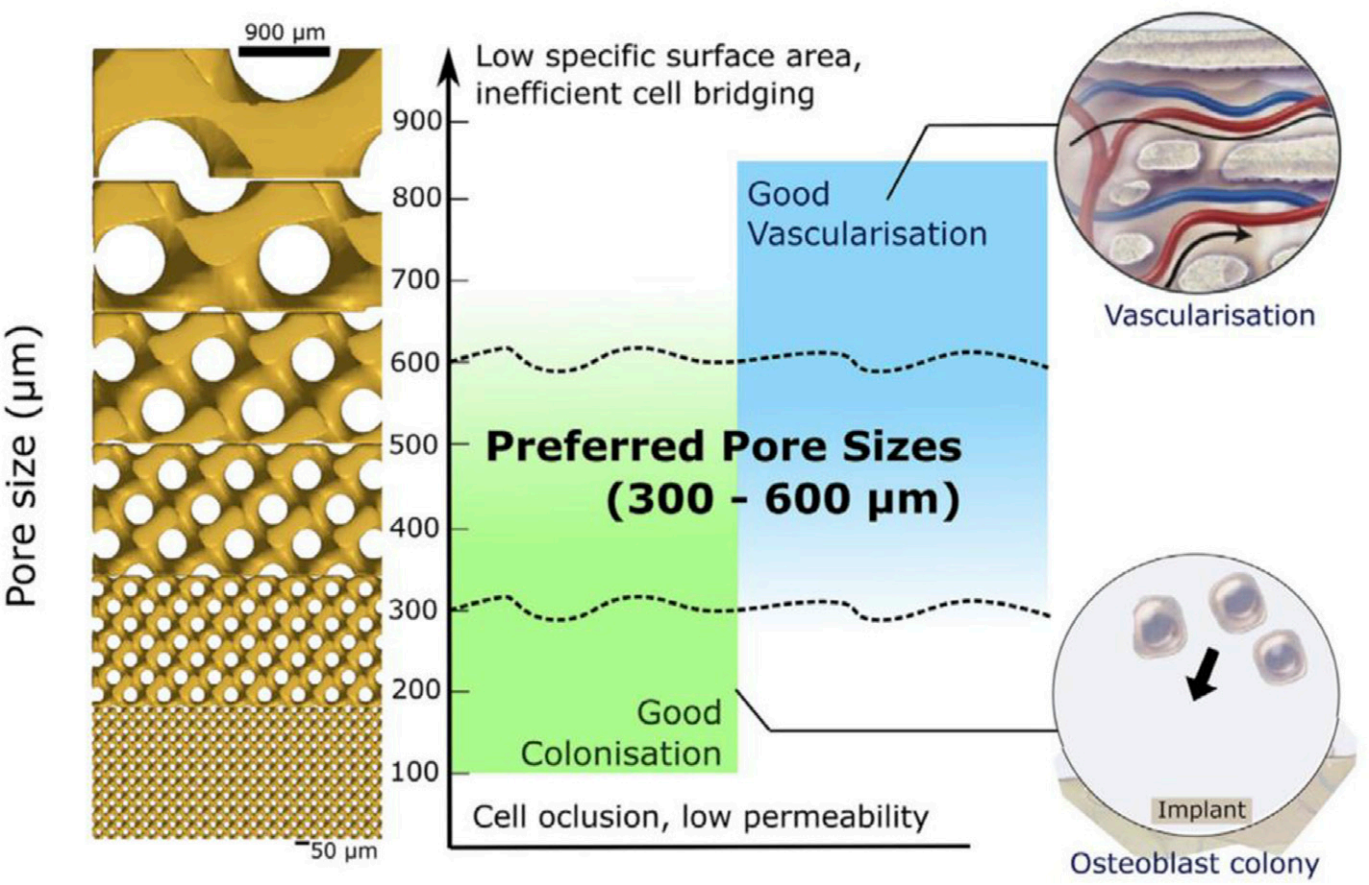
Approximate preferred regions for bone colonization and bone vascularization. Reproduced with permission (Barba et al., 2019).

Yang et al. (2017) performed finite element analysis on three types of structures: square holes, circular holes, and honeycomb holes. They found that, with the same pore diameter, the elastic modulus of honeycomb holes was the highest, followed by circular holes, and square holes had the lowest modulus. When porosity ranged from 78.4% to 89.6%, the modulus of elasticity was 21.25–13.04 GPa, which is comparable to the mechanical properties of the human femur. Molinari et al. (2018) investigated different types of porous titanium alloys produced by selective laser melting (SLM) with different geometries and porosities ranging from 47.8% to 82.6%. The results indicated that the porous structure’s strength was typically low when no struts were aligned with the load direction. The compressive strength of all specimens ranged from 10 to 250 MPa, and the modulus of elasticity ranged from 1 to 20 GPa, closely resembling the elasticity model of human cortical bone (Figure 4).

### 3.4 Clinical applications of porous titanium implants

#### 3.4.1 The advantages and disadvantages of porous titanium implants for orthopedic applications

**FIGURE 5:**
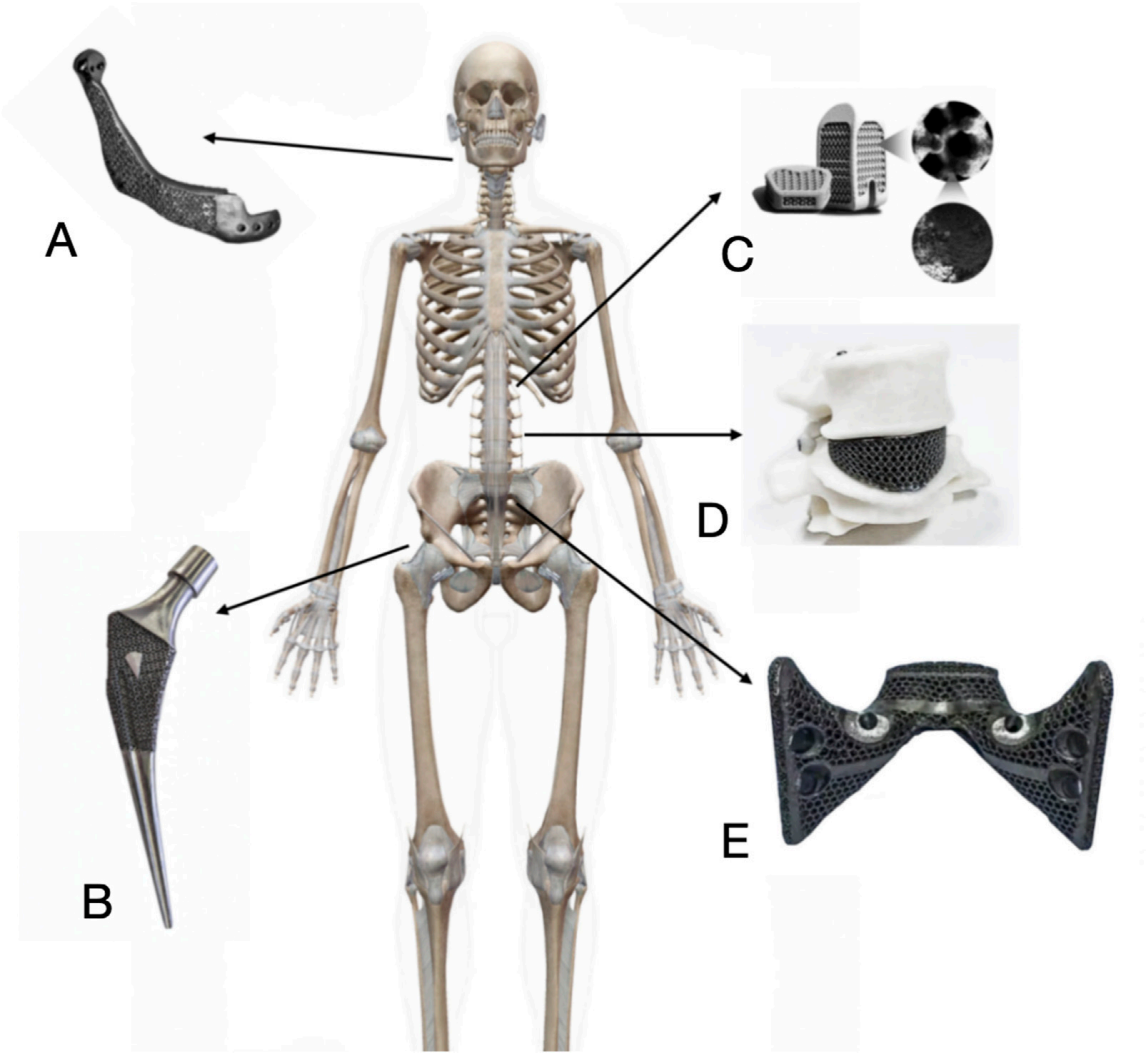
Clinical application of porous titanium alloy implants in orthopedics. **(A)** 3D print porous titanium alloy artificial mandible for mandible reconstruction after tumor resection. **(B)** Additively manufactured porous titanium alloy artificial femoral stem for hip arthroplasty. **(C)** Additively manufactured porous titanium alloy interbody fusion cage for spinal fusion surgery. **(D)** Additively manufactured porous titanium alloy artificial vertebral body for spinal reconstruction after Total *En Bloc* Spondylectomy or subtotal vertebrectomy. **(E)** Additively manufactured porous titanium alloy artificial sacrum for pelvic reconstruction after pelvic tumor surgery.

Porous titanium alloy material, with its good biocompatibility, promotes the adhesion, proliferation and differentiation of bone cells (Figure 5). Its three-dimensional connected pore structure provides favourable conditions for cell growth and nutrient transport (Nune et al., 2015). By modulating the pore parameters, the modulus of elasticity of porous titanium can be adjusted to be close to that of human bone, thereby reducing the stress shielding effect (Liu et al., 2018). The porous structure facilitates inward bone tissue growth and stable bone bonding. Surface modification techniques, such as micro-arc oxidation, further enhance the osteoinductivity of porous titanium (Nune et al., 2018). Meanwhile, additive manufacturing technology allows porous titanium implants to be personalised according to the patient’s anatomical morphology, improving the match between the implant and the defect area (Kumar et al., 2021).

However, additively manufactured porous titanium orthopaedic implants also have drawbacks. Their mechanical strength is limited, with higher porosity resulting in lower compressive strength, which may not meet the demands of highly loaded sites (Chen et al., 2017). The porous structure increases the implant’s contact area with body fluids, potentially leading to localised corrosion. Corrosion products may trigger inflammatory reactions and affect the implant’s long-term stability (Liu et al., 2012). Moreover, to improve biocompatibility and osseointegration, porous titanium implants require complex surface treatments. These processes increase manufacturing costs and time, resulting in high costs and limiting their widespread application (Wu and Guo, 2020) (Table 1).

**TABLE 1:** Clinically approved porous titanium implant system for orthopedic applications.

Diethylammonium chloride	Manufacturer	Date	Offerings	Specificities
3D printed titanium implants	Akcome Medical Care	2015	3D printed acetabular cups and patches, 3D printed spinal fusion devices and 3D printed artificial vertebrae	China’s First 3D Printed Orthopaedic Implant to Receive CFDA Marketing Approval Based on 3D ACT Technology Development
CONDUIT Intervertebral Fusion	Johnson	2017	Spinal fusion	3D printed titanium porous material with 80% porosity and elastic modulus similar to cancellous bone with increased osteoblast adhesion
Tritanium™ Titanium TL Arc Lumbar Implant	Stryker	2018	Lumbar Implants	Developed by Stryker with FDA 510(k) clearance. 3D printed using AMagine, a proprietary additive manufacturing process, with a fully interconnected porous structure that mimics the pore size and porosity of cancellous bone to promote inward bone growth and biological fixation
TiONIC™ 3D Printing Technology	Medtronic	2018	Spinal fusion	Enhanced surface textures created using differentiated laser methods to mimic natural trabecular bone morphology, increase osteoconductivity and promote osseointegration
ARTiC-L Spine System	Medtronic	2019	Spinal fusion	Using TiONIC 3D printing technology, the honeycomb structural design promotes osseointegration and improves mechanical load distribution
Hydroxyapatite-coated porous titanium interbody fusion (Wedocage™)	Professor Guo Zheng’s team at Tangdu Hospital, Air Force Military Medical University	2023	Hydroxyapatite-coated porous titanium interbody fusion device	Selected-area laser melt moulding (SLM) technology and vacuum plasma spraying technology are used to construct metal porous microstructures with bioactive surfaces. The elastic modulus of its porous structure is reduced by 37% compared with PEEK material, and the anti-sagging property reaches more than two times of PEEK fusers
FUSE TORQ TNT Pelvic Fixation Implant System	SI-BONE, INC	2024	Pelvic Implant Fixation System	3D printed porous titanium implants with specific pelvic anatomical features for fixation of pelvic fractures

#### 3.4.2 Spine

The spine, as the central axis skeleton of the human body, plays a key role in supporting body-weight and organs, protecting the spinal cord and nerves, and maintaining normal body movement. With the accelerated aging of the global population (Partridge et al., 2018) and the rapid development of transportation and manufacturing industries, spinal degeneration and trauma have become common clinical issues in spinal surgery (Otani et al., 2013). For those with cervical and lumbar spine disorders who have undergone standardized conservative treatment for 3 months or longer with unsatisfactory results, or those with obvious symptoms of limb pain who require immediate symptom relief, decompressive spinal surgery should be performed (Inose et al., 2021; Shen et al., 2018).

The interbody fusion devices used in spinal decompression have gone through several developmental milestones: autologous bone graft, where the amount of autologous bone extraction is limited while the patient needs to undergo additional iliac bone extraction surgery, which increases the surgical trauma and relevant risk (Schaaf et al., 2010); allogeneic bone graft, where the implanted allogeneic bone may trigger immune response in the patient, and there is a risk of immune rejection (Shi et al., 2014); and metal interbody fusion devices, which can provide immediate stability (Kandziora et al., 2002), but the large difference in the modulus of elasticity between metallic materials and bone leads to poor matching, insufficient strength of the bonding interface, susceptibility to complications such as stress masking, implant displacement, and vertebral body collapse (Kettler et al., 2001); non-metallic fusion devices, with the emergence of synthetic materials such as Polyether Ether Ketone (PEEK), and carbon fibers, non-metallic fusion devices have been introduced to address the drawbacks of metallic fusion devices, but the excessive dispersion of stress and high demands on the nearby vertebral endplates handling can affect the non-metallic fusion devices. However, excessive stress dispersion and high demands on the handling of the contacting vertebral endplates can affect the efficacy of nonmetallic fusion devices for spinal fusion (Kim et al., 2015).

Precision and invasiveness medicine is the emerging trends in spine surgery. In this regard, ideal surgical fusion should be achieved by placing the fusion device through a small-sized bony structure gap, with the fusion device placed in place in close contact with the vertebral bone to minimize the surgical window, reduce surgical difficulty, and improve surgical efficiency and fusion rate. Today, additively manufactured porous materials make personalized and customized interbody fusion devices possible.

The elastic modulus of traditional fusion material is higher than that of vertebral bone, and the contact between the fusion and the vertebral body will form a stress mask, and the vertebral plates nearby will undergo osteoporosis due to the lack of sufficient mechanical stimulation, which in turn will cause fusion settlement (Pan et al., 2021b). Zhang et al. (2018) compared the traditional solid metal, PEEK, and additively fabricated porous titanium based on the finite element model of lumbar interbody fusion, under different biomechanical properties under different motion modes and found that the additively manufactured porous titanium fusion significantly reduced the contact interface stresses compared to traditional solid metal or PEEK. The height of the fusion device in fusion surgery is critical to the procedure, which cannot effectively restore the intervertebral space height and Cobb angle when the height is insufficient, leading to displacement and fusion failure at the same time (Landham et al., 2017). Otherwise, it will lead to excessive intervertebral pressure between the upper and lower vertebral bodies when over-heightened, which will accelerate the fusion device sinking. Additive manufacturing allows customizing the fusion device to perfectly match the anatomical height of the intervertebral space by modeling the patient’s vertebral body with CT and MRI (Qi, 2022), while increasing the contact area and further reducing the compression by fitting the curvature and slope of the vertebral plates nearby through the fusion device’s 3D design (Adl Amini et al., 2022).

Traditional fusion devices have smooth surfaces, few hydrophilic groups, high biological inertness, and cannot provide an environment for osteoblast adhesion, resulting in low fusion efficacy (Mao et al., 2021), and clinical practice often promotes intervertebral fusion by backfilling with autogenous bone in the fusion device, while most of the bone tissues obtained intraoperatively come from the articular eminence and the vertebral plate, and the cortical bone has poor osteoblast performance and is not easy to be completely cleaned up, which may hinder affects the osteogenesis. The oxidized layer on the surface of titanium alloy can promote the binding of extracellular matrix proteins such as fibronectin and human osteosialoglycoprotein (Rapuano et al., 2012). Meanwhile, the inter-connected pore structure of porous structure, reasonably designed porosity, and rough surface can provide spatial conditions for bone tissue adhesion, proliferation, and differentiation, which is more conducive to rapid intervertebral fusion (Fogel et al., 2022) (Table 2).

**TABLE 2:** Research on the application of additive manufacturing porous titanium alloy technology in spinal surgery.

Year	Authors	Case	Type of surgery	Results	References
2016	Spetzger et al.	1	Anterior Cervical Discectomy and Fusion (ACDF)	No vertebral displacement, excellent stability	Spetzger et al. (2016)
2018	Siu et al.	1	Lumbar Interbody Fusion (LIF)	Well-matched implant endplates with lost intervertebral space height restoration	Siu et al. (2018)
2020	Arts, etc	49	ACDF	Good postoperative recovery with significant symptomatic improvement	Arts et al. (2020)
2020	Yang et al.	30	ACDF	Shorten the operation time, reduce the amount of intraoperative bleeding and the number of C-arm fluoroscopes	Yang et al. (2020a)
2021	Thayaparan et al.	1	LIF	Imaging review showed good internal fixation	Thayaparan et al. (2019)
2021	Fang et al	12	Anterior Cervical Corpectomy and Fusion (ACCF)	Good recovery of intervertebral height and cervical physiologic curvature	Fang et al. (2021)
2021	Tang et al.	27	Total *En Bloc* Spondylectomy (TES)	19 cases resumed walking, 2 cases could go outdoors with assistance	Tang et al. (2021)
2021	Liu et al.	31	ACDF	1 case of screw loosening, 1 case of implant movement, mild postoperative organic stress and axial symptoms	Liu et al. (2021)
2021	Zhang et al.	18	ACDF	Clinical efficacy	Zhang et al. (2021a)
2021	Li et al.	35	ACDF	Short operation time, low implant subsidence rate	Li et al. (2021b)
2022	Sun et al.	8	TES	No vertebral displacement, excellent stability	Sun et al. (2022)
2022	Hu et al.	8	TES	No vertebral displacement, excellent stability	Hu et al. (2022)
2022	Girolami et al	2	Anterior spinal column reconstruction	Excellent implant match	Girolami et al. (2022)
2022	Ji et al.	28	ACDF	No vertebral body displacement, precise therapeutic efficacy	Ji et al. (2022)
2023	Wang et al.	33	ACDF	Slowing of vertebral body subsidence and acceleration of intervertebral fusion	Wang et al. (2023b)

#### 3.4.3 Hip replacement

Artificial hip replacement surgery is widely employed for a wide range of severe hip diseases and is considered to be one of the most successful surgical techniques in clinical application (Gwam et al., 2017). Nevertheless, modern artificial hip replacements still face many problems including infection, wear and tear, and loosening, among which, aseptic loosening of the postoperative prosthesis is particularly prominent. Aseptic loosening is a complex process, and wear of the prosthetic material and failure of bone growth into the prosthesis are considered to be the main causes of aseptic loosening (Howard et al., 2011). Wear particles from friction of joint prostheses during use, especially ultra-high molecular weight polyethylene particles, can cause severe biological reactions, leading to periprosthetic osteolysis such as Osteoclasts abnormally activated, etc (Zaveri et al., 2017). In addition, the smooth surface of the prosthesis is not conducive to bone growth, while the difference in the modulus of elasticity between the prosthesis and the bone causes stress masking, making it difficult for remodulation of bone tissue. The prosthesis gradually develops micromotion during the process of use, which in turn triggers osteolysis and eventually leads to loosening of the prosthesis (Kröger et al., 1998).

Friction in the artificial hip joint comes mainly from the femoral head and the acetabular liner. The artificial femoral heads widely used in clinical practice are usually made of ceramic and cobalt-chromium metal alloys. Ceramic have good biocompatibility due to their material inertness, but fracture and rattling of ceramic prostheses are not uncommon (Owen et al., 2014). Cobalt-chromium alloys have excellent mechanical properties such as high strength, hardness, and elasticity, but the frictional corrosion process increases the level of metal ions in locally and systemically, sometimes leading to significant necrotic and inflammatory changes in the surrounded tissues, which in turn increases the rate of bone resorption around the prosthesis (Lohmann et al., 2013).

Titanium and its alloys are by far one of the most widely used metals within the field of medical implants. Levine (2008) have shown that porous titanium alloys are relatively close to the microstructure and properties of cancellous bone trabeculae, exhibiting good biocompatibility and mechanical properties. The long-term stability of acetabular prosthesis depends on good prosthesis-osteointegration, which determines the effectiveness of bone healing and is the key to obtaining long term stable fixation of the prosthesis, and the capacity of the prosthesis surface for bone growth and osteoinductivity is an important factor in the prevention of complications. Additively fabricated porous titanium acetabular cups allow computer design of trabecular-like structures with ideal porosity and pore size, while drugs promoting bone regeneration, such as simvastatin as a blood ester modifier (Tan et al., 2015), can be encapsulated in the porous scaffolds, further improving the osseointegration effect at the bone-prosthesis interface and enhancing the long-term stability of the hip prosthesis.

The application of additively manufactured porous titanium alloy material in hip arthroplasty is not limited to the above, however, statistics showed that there are about 35,000 patients who need to perform hip revision in the United States each year (Kurtz et al., 2007), about half of whom are accompanied by varying degrees of loss of bone mass on the acetabular side (Ulrich et al., 2008), which leads to irregular acetabular morphology and inter-individual variations. In hip revision surgery there are often large acetabular defects, and conventional metal implants and reinforcements with a single morphology are often not good enough to increase the stability of the prosthesis and affect the results of revision (Hu et al., 2013). The main principles of acetabular bone defect reconstruction are to restore the normal center of rotation of the hip joint, to preserve the original bone volume, and to obtain better initial and long-term prosthesis stability (Gao et al., 2019), and additive manufacturing can create prostheses of arbitrary shapes to accurately match with the osteotomy surfaces, and pre-position the nail channel in either direction on the prosthesis to facilitate the fixation, to restore the mechanical conduction, and to enhance the strength and stability of the bonding (Ji et al., 2013), at the same time, greatly reduce the difficulty of the surgery (Table 3).

**TABLE 3:** Research on the application of additive manufacturing porous titanium alloy technology in hip surgery.

Year	Authors	Case	Follow-up time	Results	References
2013	Colen et al.	8	10–58 months	Good postoperative recovery without periprosthetic fracture or aseptic loosening of the prosthesis	Colen et al. (2013)
2015	Mao et al.	23	Average 81.6 months	Two cases had postoperative dislocations with a mean postoperative Harris hip score of 80.9	Mao et al. (2015)
2016	Li et al.	26	Average 67 months	1 case of prosthetic loosening with a mean Harris hip score of 82 after surgery	Li et al. (2016)
2017	Wang et al.	17	Average 2 years	Postoperative Harris scores were higher in the 3D printing group than in the conventional group, but they also had higher rates of postoperative infection and prosthesis loosening	Wang et al. (2017a)
2021	Macak et al.	3	6 years	One case underwent revision surgery 18 months postoperatively, with good remaining recovery	Macák et al. (2021)
2022	Liu et al.	23	Average 51.7 months	Good mid-term clinical outcome with high acetabular survival and low rate of aseptic loosening of the prosthesis	Liu et al. (2022b)
2023	Chen et al.	11	Average 30.9 months	Good osseointegration and satisfactory short-term clinical and imaging results	Chen et al. (2023)
2024	Axenhus et al.	38	10 years	Small loss of bone density around the acetabulum improves joint replacement outcomes and implant durability	Axenhus et al. (2024)
2024	Chen et al.	236	Average 42.2 months	Excellent initial stability, high survival rate, good osseointegration, contributing to pain relief and functional improvement	Chen et al. (2024)

#### 3.4.4 Knee

The knee joint is a vital weight-bearing joint in the human body, and its integrity is essential for the daily activities and overall quality of life of individuals. In the management of bone tumors, the reconstruction of the knee joint has long been a significant clinical challenge (Fuchs et al., 2008). Traditional therapeutic approaches, such as amputation or arthrodesis, may achieve a degree of tumor control but often result in the loss of knee joint function (Pala et al., 2015). In recent years, with the advancement of material science and manufacturing technology, the application of knee joint implants in the treatment of bone tumors has made substantial progress. The development of computer-assisted technology has led to the emergence of additively manufactured titanium porous scaffolds as a novel structural option for orthopedic implants. Additive manufacturing technology offers controllable and precise fabrication processes, enabling the production of individualized implants tailored to the specific needs of patients. These porous implants can be designed to match the structure of human bones by adjusting porosity, thereby mitigating the stress shielding effect often associated with implants. Moreover, the porous surface of the implants provides ample space to promote bone tissue growth and enhance the stability of the bone-prosthesis interface.

Liu et al. (2020) conducted a follow-up study on 12 patients with malignant bone tumors in the metaphyseal region around the knee joint from September 2016 to October 2018. These patients underwent joint-sparing mesenchymal tissue resection surgery. Utilizing preoperative CT and MRI data, computer-assisted design technology was employed to create three-dimensional models and design individualized osteotomy guides and prostheses. Accurate osteotomy was performed using 3D-printed osteotomy guides, followed by the implantation of 3D-printed prostheses that were precisely matched to the residual bone. The fixation methods for the prostheses were selected based on the patients’ age and bone maturity. The average follow-up duration postoperatively was 22.5 months. Tumor tissues were accurately resected in all patients, with a good match between the residual bone and the prosthesis, with discrepancies within 2 mm. The average postoperative Musculoskeletal Tumor Society (MSTS) score was 28 (range 26–30), and 10 patients achieved satisfactory knee joint range of motion (average flexion 108°, range 90°–125°). All patients were able to ambulate without assistance. Two patients experienced superficial infections; however, there were no deep infections or prosthesis-related complications. One patient had local soft tissue recurrence, but the recurrent tumor was distant from the metaphyseal resection plane; another patient developed lung metastasis 15 months after surgery. The use of individualized osteotomy guides and prostheses effectively facilitated accurate tumor resection and functional reconstruction while preserving the joint. The surgical outcomes were favorable, with satisfactory postoperative functional recovery and a low complication rate.

#### 3.4.5 Shoulder

Shoulder arthroplasty is primarily employed to address severe shoulder pathologies, including complex proximal humerus fractures, severe osteoarthritis, rheumatoid arthritis, and shoulder joint bone tumours in elderly patients (Reuther et al., 2010). Common postoperative complications encompass joint instability, infections, prosthesis loosening, periprosthetic fractures, and sports injuries, which are frequently implicated as a cause of shoulder arthroplasty failure (Fialka et al., 2008). The intricate anatomical structure of the shoulder joint presents challenges in matching bone defects during shoulder arthroplasty or humeral tumour resection with conventional shoulder prostheses (Weng et al., 2016). Moreover, the absence of tendon stops and soft tissue laxity in the rotator cuff can lead to postoperative shoulder instability or even dislocation. However, the advent of additive manufacturing technology has provided novel opportunities to address these challenges.

Zou et al. (2018) reported a case of shoulder arthroplasty revision utilizing a custom 3D-printed porous titanium shoulder implant. The patient had undergone proximal humeral resection and shoulder arthroplasty for chondrosarcoma of the shoulder. However, due to severe bone defects resulting from implant loosening, conventional implants were deemed inadequate for revision surgery. By leveraging additive manufacturing technology and computer-aided design, a customised porous titanium implant was successfully fabricated. This implant effectively reconstructed the anatomical structures and demonstrated satisfactory functional recovery and bone healing during postoperative follow-up. The study highlighted that customised porous titanium implants offer significant advantages in managing complex bone defects, providing superior anatomical fit and functional outcomes.

#### 3.4.6 Ankle

The ankle joint, characterized by its complex structure and significant load-bearing capacity, plays a crucial role in human locomotion. The primary therapeutic objective for treating ankle joint diseases and injuries is to restore the joint’s anatomical integrity and functionality while minimizing complications. Reconstruction following complex skeletal defects and resection of malignant tumors in the ankle remains a formidable challenge in the field of orthopedics (Nauth et al., 2018). Traditional treatment modalities, such as autologous and allogeneic bone grafting, are often associated with drawbacks, including donor site morbidity, infection risk, and the potential for non-union (Blair et al., 2015).

Abar et al. (2022) conducted a retrospective study on patients who received custom additive-manufactured titanium ankle implants between 1 June 2014, and 30 September 2019. The study revealed that 74% of patients did not require subsequent surgical intervention following the implantation of custom 3D-printed titanium implants. Moreover, the need for secondary surgery was significantly correlated with the presence of neuropathy. This investigation represents the most extensive study to date validating the efficacy of custom titanium ankle implants. Wang et al. (2025) reported a case in which additive manufacturing technology was utilized to fabricate an ultra-high molecular weight polyethylene-porous titanium calcaneal prosthesis for the treatment of ankle Ewing’s sarcoma. At the 12-month follow-up, the patient exhibited satisfactory wound healing and substantial pain relief. The range of motion of the ankle joint was measured at 10° for dorsiflexion, 35° for plantarflexion, 15° for inversion, and 10° for eversion. Radiographic evaluation demonstrated optimal prosthesis positioning, and T-SMART technology confirmed robust bone-prosthesis integration. Notably, no implant-related complications were observed within the 12-month postoperative period. Collectively, these findings underscore the promising outcomes of additive manufacturing porous titanium ankle implants in terms of postoperative functional recovery and bone integration.

#### 3.4.7 Bone related tumors

Since the development of additively manufactured porous titanium alloys, a number of personalized implants and related anatomical models have been designed and fabricated for prosthetic reconstruction after resection of bone tumors. The principle of tumor treatment is complete and extensive resection, and bone tumors are highly challenging due to their complex anatomy and lack of reconstructive scaffolds. When faced with extensive and irregular bone defects after tumor resection, autologous bone grafts or allografts often fail to meet the clinical needs, and additively fabricated porous titanium scaffolds can be a good solution to this problem.

It has been reported that additively fabricated porous titanium alloy materials have shown promising efficacy in the surgical treatment of multiple sites and types of tumor invasion. Specifically, the material has been successfully applied in surgery for different types of tumors and invasion sites, such as atlantoaxial vertebrae infiltrated by Ewing’s sarcoma (Xu N. et al., 2016), vertebrae infiltrated by chordoma (Yang, 2016), iliac bone infiltrated by chondrosarcoma (Ye et al., 2015), and heel bone infiltrated by mesenchymal fibroma (Park et al., 2018). These studies have demonstrated that porous titanium alloy materials not only provide the necessary structural support, but also promote osseointegration and functional recovery, improving patients’ postoperative quality of life.

Patients with bone related tumors (sarcoma or bone metastasis) often need to receive systemic chemotherapy after the completion of bone defect repair and reconstruction in order to avoid tumor residue and recurrence as much as possible. The systemic use of chemotherapeutic drugs not only produces huge toxic side effects, but also the concentration of anti-tumor drugs at the site of tumorigenesis often fails to reach the standard due to the impaired blood supply. Porous titanium alloy implant has a unique porous structure. By modifying the surface of the implant or loading its internal space with anti-tumor drugs for controlled release, it can sustainably inhibit the proliferation of tumor cells and induce apoptosis of tumor cells to achieve the effect of local chemotherapy.

Studies have shown that hyaluronic acid-based nanoparticles can be used for the reversal of immune suppression and immunochemotherapy in osteosarcoma treatment. These nanoparticles can target the delivery of doxorubicin, cisplatin, and resiquimod, and release the drugs in the acidic tumor microenvironment, inducing tumor cell apoptosis, and triggering immunogenic cell death. They could also promote the presentation of tumor-associated antigens and the induction of anti-tumor immunity, which would significantly inhibiting tumor growth and lung metastasis (Zhang et al., 2021b), and the inhibitory effect on the proliferation of cancer cells is much greater than that to normal cells (Han et al., 2014). Zhang et al. (2019) prepared porous titanium alloy scaffolds by laser sintering, and then prepared n-HA coating on the surface of the porous titanium alloy scaffolds by slurry foaming method, and then implanted them into a rabbit femur tumor resection post-defect model. The results showed that the n-HA-loaded porous titanium alloy scaffolds could effectively prevent tumor metastasis, and the n-HA coating promoted the regeneration of new bone within the pores of the porous titanium alloy scaffolds while inhibiting the growth of tumors.

Magnesium and its alloys are biodegradable materials that not only promote bone regeneration at defects, but their degradation products also show good anti-tumor properties *in vitro* and *in vivo*. Meanwhile, the degradation cycle of magnesium *in vivo* can be adjusted to vary from half a year to several years, which can fight against bone tumor cells *in vivo* for a long period of time (Zhang et al., 2019). Wei et al. (2021) prepared porous titanium alloy scaffolds by using additive manufacturing technology, and magnesium coatings on the surface of the scaffolds were prepared by using multi-arc ion plating technology. The results showed that the implant released magnesium ions during magnesium degradation, and the appropriate concentration of magnesium ions could effectively inhibit the proliferation of bone tumor cells and increase the apoptosis of tumor cells.

In recent years, researchers have paid attention to the possibility of embedding antitumor drugs in porous titanium alloy implants. Jing et al. (2021) utilized porous titanium alloys as substrate scaffolds and injected hydrogels containing the antitumor drug cisplatin into these porous structures. It was found that this porous titanium alloy implant incorporated with cisplatin hydrogel showed significant anti-tumor effects both *in vitro* and *in vivo*. In particular, in a murine tumor model, local release of cisplatin through the implant not only reduced adverse effects but also enhanced anti-tumor effects compared to systemic cisplatin treatment. In addition, the integrative effect of this composite scaffold with the surrounding bone tissue was validated by assessing the index of inward bone growth (Table 4).

**TABLE 4:** Research on the application of additive manufacturing porous titanium alloy technology in bone tumor surgery.

Year	Authors	Case	Tumor type	Location	Follow-up time	Results	References
2015	Fan et al.	3	Ewing sarcoma in 2 cases, osteosarcoma in 1 case	Clavicle, scapula, pelvis	2 years	No tumor recurrence, periprosthetic fracture, aseptic loosening of prosthesis	Fan et al. (2015)
2016	Xu et al.	1	Ewing sarcoma	C2-T2	1 year	No tumor recurrence, vertebral body displacement, postoperative JOA score of 16	Xu J. et al. (2016)
2017	Liang et al.	12	Pelvic tumor	Pelvic	Average 20.5 months	Well integrated and stable prosthesis	Liang et al. (2017)
2017	Luo et al.	4	Giant cell tumor	Tibial	Average July	No tumor recurrence, periprosthetic fracture, aseptic loosening of prosthesis	Luo et al. (2017)
2017	Kim et al.	1	Osteosarcoma (benign tumor composed of bone-like material)	Bone forming the base of the spinal column	1 year	Good integration, no tumor recurrence, vertebral body displacement, postoperative VAS score of 3	Kim et al. (2017)
2017	Li et al.	1	Thyroid cancer spinal metastasis	C2-C4	1 year	No tumor recurrence, vertebral body displacement, postoperative JOA score of 11	Li et al. (2017)
2018	Lu et al.	1	Osteosarcoma (benign tumor composed of bone-like material)	Tibial	Average 2 years	No tumor recurrence, periprosthetic fracture, aseptic loosening of prosthesis	Lu et al. (2018)
2019	Feng et al.	1	Adenoblastoma	Extremities	1 year	Well integrated and stable prosthesis	Feng et al. (2019)
2019	Lu et al.	1	Giant cell tumor	Tibial	29th month	No tumor recurrence, periprosthetic fracture, aseptic loosening of prosthesis	Lu et al. (2019)
2019	Han et al.	1	Metastatic renal clear cell carcinoma	Pelvic	1 year	No tumor recurrence, periprosthetic fracture, aseptic loosening of prosthesis	Han et al. (2019)
2020	Chen et al.	1	Pelvic tumor	Pelvic	1 year	Significant pain relief and good functional recovery	Chen et al. (2020a)
2020	Wei et al.	9	Primary spinal bone tumors	Columna vertebralis	Average 28.6 months	2 cases of tumor recurrence and metastasis, no tumor recurrence and vertebral body displacement in the remaining cases	Wei et al. (2020)
2020	Yang et al.	1	Chordoma	C2-T2	September	No tumor recurrence, vertebral displacement	Yang et al. (2020b)
2020	Wu et al.	1	Giant cell tumor	L4	13th month	No tumor recurrence, vertebral displacement	Wu et al. (2020)
2020	Li et al.	1	Atlantoaxial isolated plasma cell	C1	1 week	No tumor recurrence, vertebral displacement	Li et al. (2020)
2020	Zhao et al.	5	2 cases of osteosarcoma, 1 case of Ewing’s sarcoma, 1 case of pseudomyxoid-derived hemangioendothelioma, 1 case of undifferentiated pleomorphic sarcoma	Tibial	Average 27.6 months	No tumor recurrence, periprosthetic fracture, aseptic loosening of the prosthesis, and a mean postoperative MSTS score of 26.8	Zhao et al. (2020)
2021	Wu et al.	28	Pelvic tumor	Pelvic	Average 32.2 months	No tumor recurrence, periprosthetic fracture, aseptic loosening of the prosthesis, and a mean postoperative MSTS score of 23.2	Wu et al. (2021)
2021	Wang et al.	1	Breast cancer spinal metastasis	T11-L1	2 years	No tumor recurrence, vertebral displacement	Wang et al. (2021a)
2021	Wang et al.	15	Osteosarcoma 10, chondrosarcoma 2, Ewing’s sarcoma 3	Femur, tibia	Average of 42 months	1 case of aseptic loosening of the prosthesis, and the remaining postoperative MSTS score averaged 26 points	Wang et al. (2021b)
2021	Zhang et al.	8	Giant cell tumor	Tibial	Average of 26 months	No tumor recurrence, periprosthetic fracture, aseptic loosening of prosthesis	Zhang et al. (2021c)
2022	Park et al.	12	Pelvic tumor	Pelvic	—	One case was amputated 4 months after surgery due to local recurrence	Park et al. (2021)
2022	Zhou et al.	23	Thoracolumbar spinal tumors	Columna vertebralis	Average 37 months	No tumor recurrence, vertebral displacement	Zhou et al. (2022)

### 3.5 Post-processing of porous titanium implants

Bone defect repair has always been one of the challenging topics in orthopedic clinics, and with the development of additive manufacturing technology and material science, accumulating advanced bone tissue engineering scaffolds with matched mechanical strength and biomimetic structures have been developed to meet the increasing demand for bone repair (Maresca et al., 2023).

Titanium and its alloys are well suited as bone tissue substitutes due to their excellent biocompatibility, outstanding corrosion resistance and low tissue responsiveness (Xu et al., 2022). However, poor adhesion between the implant-bone interface and stress shielding due to high stiffness remain major drawbacks of titanium implants (Kapat et al., 2017).

In recent years, the development of implant surface modification technology has provided new ideas to solve the above problems. By changing the surface morphology or modificating implant surface, in order to achieve anti-infection capacity, promote osteogenesis, optimize wear resistance, corrosion resistance and oxidation resistance. It has been demonstrated that physical and chemical-based surface modifications can influence cellular behavior (Sheng et al., 2022; Li et al., 2021c), while immobilization of biomolecules on the implant surface and local delivery of biotyping molecules at appropriate concentrations and rates can directly influence biochemical processes and effectively induce bone formation (Lutz et al., 2010; Le Nihouannen et al., 2008).

In addition, by applying surface coatings made of biomolecules with well-defined bioactivities, it is possible to tailor the biochemical properties of the implant surface to more effectively modulate the interactions between the biomaterials and the implanting microenvironment (Sobolev et al., 2019), to directionally regulate the adhesion behavior of specific cells, to influence their differentiation process, to shorten the healing cycle, and to enhance the long term stability of the implant (Villegas et al., 2022).

#### 3.5.1 Surface modification

After the implant is placed into the body, its surface will have a direct contact effect with the tissue cells in the implanted environment, so the physicochemical properties of the implant surface are the key factors affecting the implantation success, stability, and longevity of the implant (Mendonça et al., 2008). In order to enhance the biocompatibility of titanium alloy implants, improve their stability *in vivo*, and further shorten the healing time, surface modification of the implant is required prior to implantation. Studies have shown that surfaces with micro- and nanocomposite structures can promote the proliferation and differentiation of osteoblasts (Hori et al., 2010), while the combination of nanoscale and microscale surface features that mimic the graded structure of bone can further improve the bone growth-promoting properties of implants and shorten the healing time (Gittens et al., 2011).

##### 3.5.1.1 Chemical treatment

The surface of titanium alloys is susceptible to corrosion by chemicals and strong acids, and this corrosive effect changes the surface texture and roughness of the material. And the Topological structure of surface is a key determinant of biological processes such as adsorption, movement and differentiation of, proteins, bacteria and cells, respectively (Surmeneva et al., 2022). Civantos et al. (2019) investigated the effect of hydrofluoric acid etching time on the *in vitro* cellular behavior of powder sintered porous titanium scaffolds. By etching the scaffolds, the average surface roughness of the material gradually increased with etching time. *In vitro* experiments revealed that the surface characteristics not only affected osteoblast adhesion, but also promoted osteoblast differentiation by increasing the production of bone matrix proteins, including ALP, collagen, osteoblastin, and osteocalcin. However, etching improves the surface structure of the material while causing a decrease in the strength of the material (Wang et al., 2020a), while the surface morphology is difficult to control and may cause damage to the material, limiting its application.

Physical, chemical impregnation and deposition is a method of forming coatings on the surface of porous metallic materials by impregnation, deposition or chemical reaction after mixing uniformly in the liquid phase with bioactive inorganic salts such as hydroxyapatite or organic compounds such as polydopamine, peptides, amino acids, and so on as the precursors. Yuan et al. (2022) used deposition to coat hydroxyapatite particles on the surface of porous titanium scaffolds, and *in vitro* cellular assays showed that it induced no statistically significant increase in hFOB1.19 apoptosis, and the hydroxyapatite-coated group presented higher CCK-8 values compared to the control group. Wang et al. (2010) immersed porous titanium alloy scaffolds into sodium hydroxide solution for heating treatment and then immersed them in simulated body fluids for 1 week to make the calcium phosphate elements uniformly deposited on the surface of the scaffolds. Cell culture experiments demonstrated that the synthetic material significantly enhanced the osteointegration effect and enhanced the phenotypic osteogenic profile of mesenchymal cells and osteoblast synthesis activity. Wu (2019) immersed porous titanium alloy in alkaline polydopamine solution to form a coating of a certain thickness on the surface of the material through polydopamine self-polymerization reaction. Bio-mineralization experiments showed that the phenolic hydroxyl groups in polydopamine interact with calcium ions, which can provide more nucleation sites and improve the surface bio-mineralization properties. In addition to polydopamine, proteins such as extracellular matrix proteins, growth factors, polysaccharides and nucleotides have also been used for bioactive organic modifications (Che et al., 2022), however, some organic coatings are highly targeted in antimicrobial and osteogenic promotion. However, inorganic coatings such as hyaluronic acid do not bind well to the material and tend to break off under cyclic loading, affecting their therapeutic effect.

Electrochemical method is to utilize metal materials as electrodes in the electrolyte, through redox reaction on the surface of the material to form porous oxides or coatings containing specific components. According to the type of reaction and the principle of action is mainly divided into anodic oxidation, micro-arc oxidation, electrochemical deposition process.

Anodizing is a method of building oxide film, corrosion resistant pits and titanium nanotubes on the surface of the scaffold by the action of external current using titanium or its alloys as an anode and placed in an electrolyte of hydrofluoric acid system. Shokuhfar et al. (2014) anodized the surface of the material using tetrahydrofuran solution as an electrolyte and explored its biological properties. Osteoblast culture experiments showed that the presence of nanotubes increased the total cell density with the same matrix material.

Microarc oxidation is to increase the voltage or current on the basis of anodic oxidation, using the strong voltage to generate microarc discharge and local high temperature, so that the electrolyte ions in the discharge channel are vaporized at high temperature, forming plasma and oxidizing with porous implants, thus generating porous oxide coatings with titanium oxide as the main constituent (Li et al., 2023b). Luo et al. (2021) used a calcium acetate electrolyte solution for porous titanium alloy implants were subjected to micro-arc oxidation. A micro and nanostructure-containing layer with a thickness of 6–7 μm was formed on the material surface. It was found that Calcium in the coating and the porous structure significantly enhanced cell viability and increased the formation of new bone *in vivo*. Compared with anodic oxidation, the coatings prepared by microarc oxidation had high hardness, high bond strength and good wear resistance.

**FIGURE 6:**
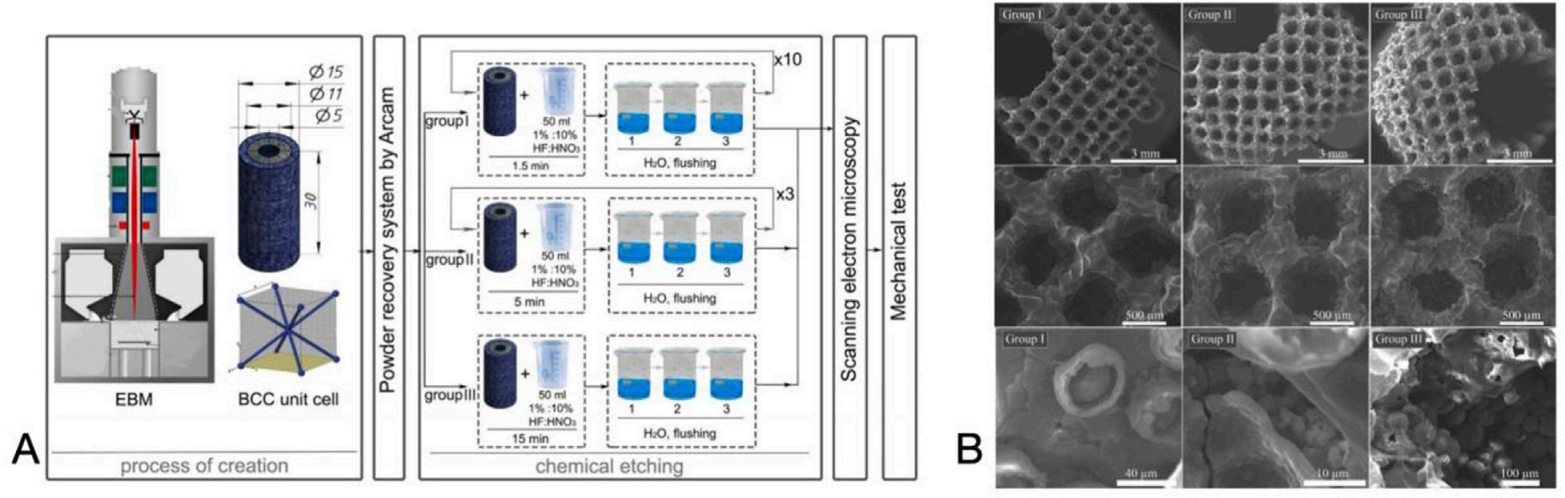
**(A)** Preparation of the Scaffolds and the Chemical Etching Process. **(B)** SEM images of the surface layer formed on the scaffold. Reproduced with permission (Surmeneva et al., 2022).

Electrochemical deposition, also known as electrodeposition, electrophoretic deposition, or electroplating, is a method of depositing materials (metals, polymers, ceramics, glass, and their composites) onto a substrate material using an electric current through a redox reaction (Weng et al., 2017). Vidal et al. (2021) performed pulsed electrodeposition of porous titanium alloy scaffolds prepared by ink-direct technology to achieve a uniform coverage of the Ca-P coating. The results of antimicrobial tests showed that the biofunctionalized scaffolds displayed antimicrobial activity against Gram-positive and Gram-negative strains, effectively improving the biointegration properties of orthopaedic implants while reducing peri-implant infections. In addition, researchers plated Cu, Zn, Ag, Mg, and Ta onto the material surface to enhance wear and corrosion resistance and biocompatibility (Fan et al., 2022b). Electrochemical deposition produces coating materials with better uniformity, a wider range of thicknesses, and higher bond strengths than traditional chemical deposition (Figure 6).

##### 3.5.1.2 Metallic oxide coatings

Metal oxide coatings, as a crucial surface modification technique, markedly enhance the wear resistance, corrosion resistance, and high-temperature oxidation resistance of metal materials. Current preparation methods primarily include vapour phase deposition, micro-arc oxidation, and plasma electrolytic oxidation.

Vapour phase deposition involves the gasification of components to be added, which then contact porous titanium to form a thin film on the surface. This process can be categorized into physical vapour phase deposition (PVD) and chemical vapour phase deposition (CVD) based on the gasification principle. PVD is a technique where the target material migrates and accumulates on the substrate surface in atomic, molecular, or ionic form through evaporation or sputtering in a vacuum environment, forming a thin film. Its main types include vacuum evaporation, sputtering coating, and ion plating (Wang et al., 2017b). Diez-Escudero et al. (2022) applied PVD to deposit silver coatings on porous Ti6Al4V alloys, with a thickness of (4.5 ± 1.5) μm. Compared to the blank group, these coatings reduced the adhesion of *Staphylococcus aureus* and inhibited biofilm formation on the material surface within 72 h. CVD involves depositing metallic or inorganic coatings on a substrate by vaporizing compounds containing the elements of the thin film and chemically reacting them with the substrate surface (Cui et al., 2022). Lascano et al. (2020) used CVD to transfer a monolayer of graphene onto porous titanium alloy surfaces, enhancing cellular adhesion and proliferation. Additionally, the graphene layer acts as a barrier, reducing cell contact with residual metallic heavy elements in the alloy.

**FIGURE 7:**
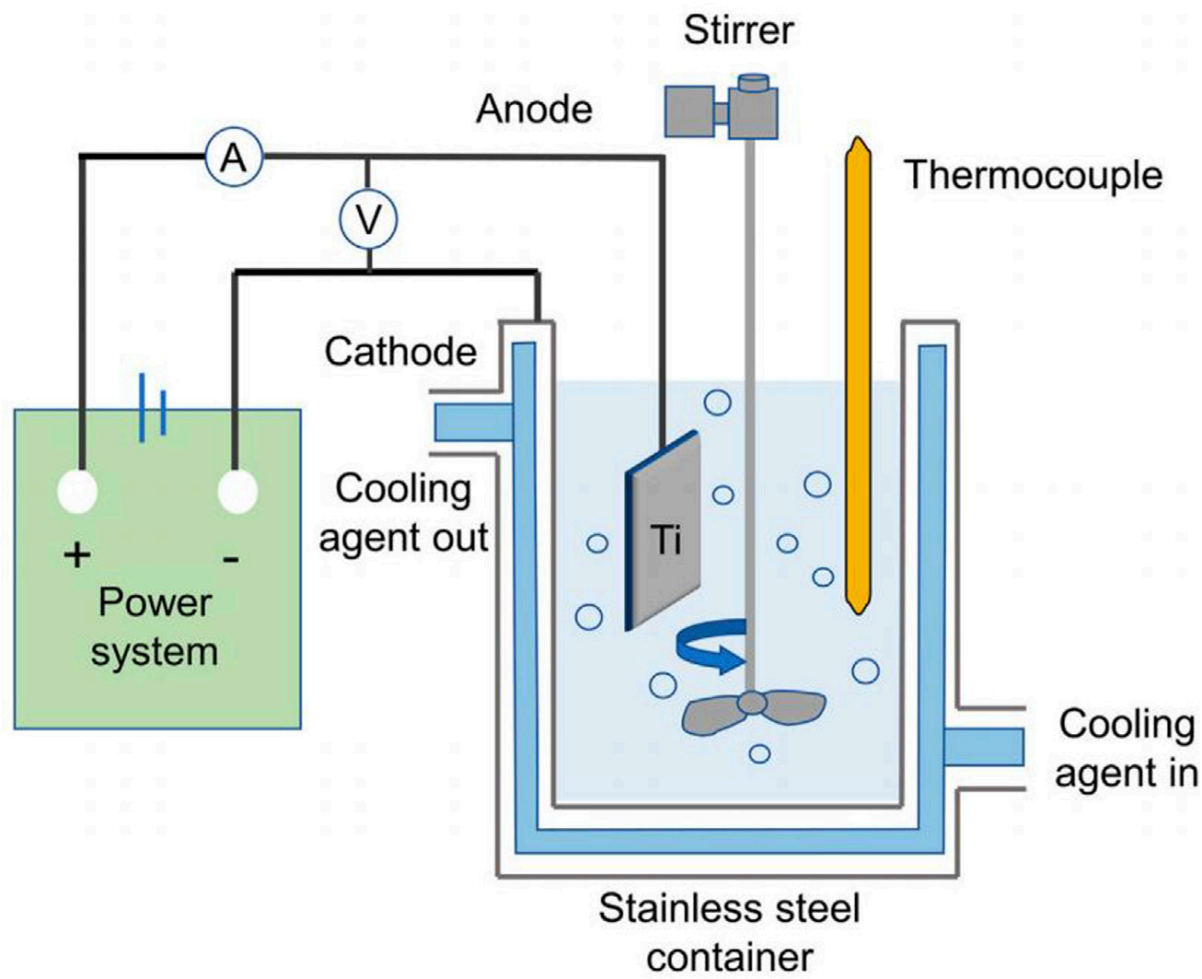
Schematic representation of the MAO coating system. Reproduced with permission (Wen et al., 2023).

Micro-arc oxidation (MAO) is a surface modification technique derived from anodizing. It involves immersing metal in an alkaline electrolyte and inducing micro-discharge on the metal surface via high voltage. The local high temperature generated by the discharge promotes oxidation of the base metal, forming a complex oxide ceramic coating dominated by substrate element oxides, with electrolyte components participating in doping modification (Rakoch et al., 2006). The coating formation mechanism, involving breakdown-channeling-melting effects, has been extensively studied. MAO coatings exhibit superior wear resistance, corrosion resistance, and fatigue resistance (Zhang et al., 2023). Additionally, MAO treatment enhances the cytocompatibility of titanium and its alloys, promoting cell adhesion, proliferation, and differentiation (Wen et al., 2023). However, the potential risk of bacterial infections in medical applications can be mitigated by incorporating bio-antimicrobial agents and metallic elements such as Cu, Ag, Zn, and Mg into the MAO coatings (Huang et al., 2021) (Figure 7).

Plasma electrolytic oxidation (PEO) is a surface treatment method that integrates electrochemical oxidation with plasma physics. By applying high-voltage pulses in an electrolyte solution, PEO induces plasma discharge on the metal surface to form a ceramic oxide coating (Hussein et al., 2013). In recent years, PEO has garnered significant attention due to its simple process, low cost, and superior coating properties. This method can create coatings on substrates with diverse geometries, enhancing bond strength. On titanium-based alloys, PEO can be performed with various electrolytes to produce calcium phosphate-based composite layers with distinct morphology, structure, thickness, and crystallinity (Rafieerad et al., 2015). These coatings typically exhibit hardness values above 1000 HV, significantly exceeding that of the base metal. Moreover, the wear resistance of PEO coatings can be further optimized through process parameter adjustments (Lederer et al., 2021).

##### 3.5.1.3 Inorganic coatings

The development of titanium alloy laser melting surface modification technology has progressed from laser surface quenching to laser surface remelting, laser surface synthesis, and ultimately to laser cladding (Zhao et al., 2023). Laser cladding entails spraying premixed powder onto the substrate surface via a nozzle, and forming a molten pool by adjusting laser power, spot size, and scanning speed. By varying the powder composition and continuously spraying into the pool, a functionally gradient coating is achieved (Song and Li, 2010).

**FIGURE 8:**
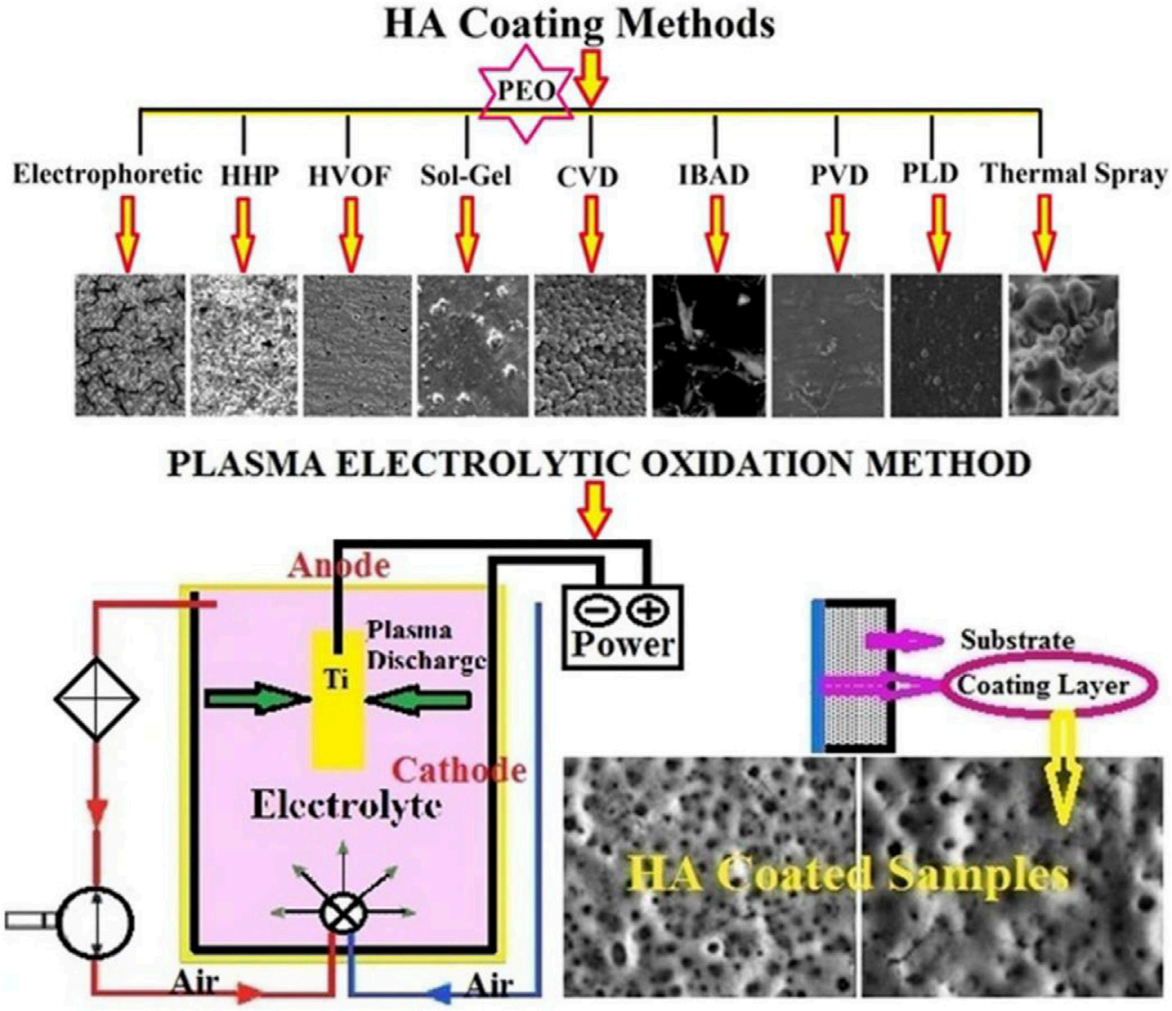
Morphology of the HA layer via different coating methods and schematic view of PEO method. Reproduced with permission (Rafieerad et al., 2015).

Laser melting and cladding technology integrates the benefits of arc and electron beam melting, characterized by high energy density and environmental adaptability (Ouyang et al., 2022). It enables the deposition of thin layers on complex structural surfaces using powders or filaments. Combined with small laser spot diameters, the cladding layer thickness, surface roughness, and layer interfaces can be optimized through parameter adjustments (Wang et al., 2021c). These features facilitate the formation of metallurgically bonded cladding layers with superior mechanical properties, thereby enhancing coating quality (Arif et al., 2021). While laser cladding for thin film preparation is rapid, adaptable, and highly controlled, it is also associated with high equipment costs and process complexity (Figure 8).

#### 3.5.2 Hydrogel

Hydrogels are soft polymers with a three-dimensional mesh structure formed by cross-linking of monomers or polymer chains through covalent or non-covalent bonding interactions (e.g., hydrogen bonding, electrostatic interactions, host-guest complexation, and their combinations) (Ali et al., 2019). Hydrogels can be prepared from natural (e.g., filipin proteins, chitosan, and gelatin) or synthetic polymers [e.g., poly (vinyl alcohol), poly (ethylene glycol), and poly (betaine)] (Lee et al., 2013). Hydrogels composed of polysaccharides and proteins possess physical and chemical properties similar to those of most human tissue (Chen et al., 2019a). Due to the softness and wettability that hydrogels possess, they are very similar to the extracellular matrix (ECM) of biological tissues and can provide ideal ecological conditions for cell survival (Tibbitt and Anseth, 2009).

With the aging of the population in society, osteoporosis has become a common disease endangering the life and health of middle-aged and elderly people, usually seen in postmenopausal people aged over 65 years, which is characterized by a decrease in bone mineral density due to bone loss accompanied by an increase in bone fragility and sensitivity (Cruz et al., 2018). The treatment of osteoporotic bone defects is a complex clinical problem, and due to the bioinertness of titanium and its alloys and the fact that in the setting of osteoporotic pathology, although porous scaffolds facilitate bone growth into the scaffolds, bone regeneration is severely limited and implants may struggle to achieve good osseointegration. Therefore, it is necessary to stimulate bone regeneration to achieve better bone restore.

Silk proteins are natural protein with excellent biocompatibility and good mechanical properties make them ideal candidates for bone regeneration promotion (Tan et al., 2005). Silk proteins have been processed into various forms such as fibers, hydrogels, scaffolds, films, sponges, and microspheres through different techniques (e.g., electrostatic spinning, 3D printing, and freeze-drying) (Grigoryan et al., 2019). Among them, hydrogels are one of the important forms of silk protein-based materials. Silk protein hydrogels can be prepared by a variety of methods, and the prepared hydrogels have numerous tunable properties, and have been widely used in the field of medicine due to the advantages of mild preparation conditions, controllable reaction time, and reduced by-products (Pal et al., 2014).

Studies have shown that filipin protein itself can improve the interaction between osteoblasts and fibroblast biomaterials (Xu et al., 2020). In order to improve the integration of titanium implants with the surrounding bone tissue, Saha et al. (2019) coated the surface of titanium implants with filipin protein, and it was found that filipin protein enhanced osteoconductivity and osteoblastogenic properties. Lee et al. (2017) prepared a physically crosslinked filipin protein and poly (vinyl alcohol) by a high-strength filipin protein hydrogel, which had mechanical properties similar to those of human ear cartilage, and the hydrogel maintained its original shape throughout the culture process. Histological analysis showed that the prepared hydrogel had excellent biocompatibility and provided a good environment for chondrocyte adhesion and proliferation.

Scaffolds for clinical applications in bone tissue engineering not only need to have the ability to promote the generation of new bone, but they must also contain interconnected vascular networks to facilitate nutrient transport, oxygen exchange, metabolic waste elimination, and regulation of cells and signaling molecules (Wan et al., 2020). Therefore, when designing bone graft replacement materials, both osteogenesis and angiogenesis must be induced (Barati et al., 2016). During bone restoring, vascular endothelial growth factor (VGEF) secreted by osteoblasts promotes the proliferation and migration of endothelial cells, while VEGF also increases the level of bone morphogenetic protein (BMP) in endothelial cells. Endothelial cells in turn secrete osteogenic factors, such as BMP-2 and BMP-4, to enhance osteoblast differentiation. Thus, it is clear that angiogenesis and osteogenesis are closely linked (Zhang et al., 2022a). Numerous studies have demonstrated the synergistic effect of angiogenic factors with osteogenesis-related factors (Bosetti et al., 2007; Liu et al., 2007; Izquierdo-Barba et al., 2019), which have also been used in combination with implants. For example, Schorn et al. (2017) developed an implant that consisted of a collagen scaffold containing BMP-2 and VEGF. Patel et al. (Orth et al., 2022) devised a method of mixing VEGF and BMP-2 into gelatin particles and implanting these particles into porous polypropylene fumarate scaffolds for use in a rat cranial defect model. Despite the effectiveness of these methods for bone regeneration, these scaffolds were unable to maximize osteogenesis because they were unable to control the release of VEGF and BMP-2. For this reason, Lv et al. (2015) combined porous titanium implants with fibrin glue containing growth factors to form a composite porous titanium scaffold. Fibrin was used as a carrier to control the release of BMP-2 and VEGF. Preliminary histologic evaluation showed that the incorporation of growth factors significantly promoted the formation of new bone tissue in the vasculature and stent pores.

**FIGURE 9:**
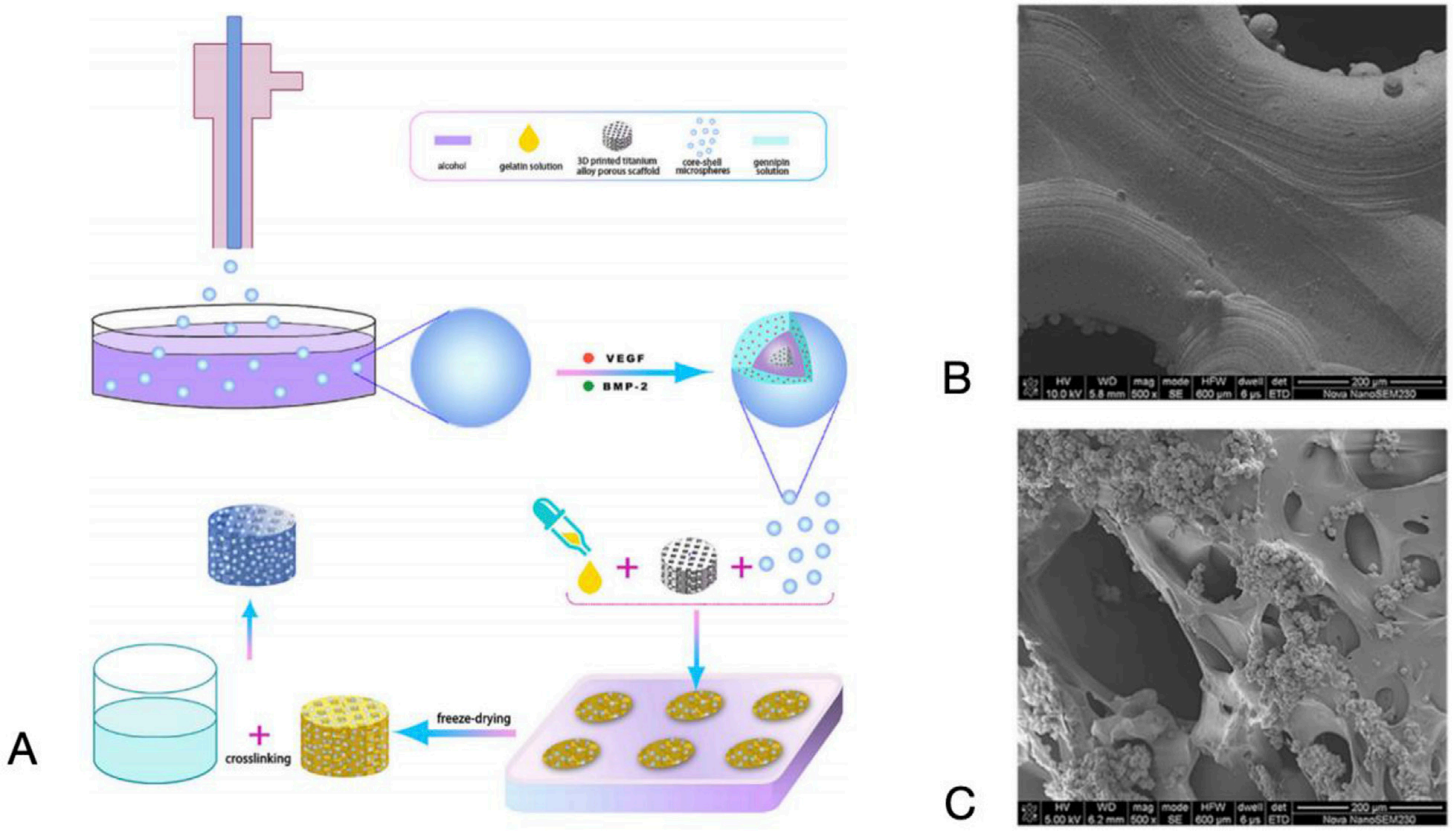
**(A)** Schematic diagram of the preparation process of the composite scaffold system. **(B)** SEM images of titanium alloy porous scaffolds. **(C)** Dual-factor loaded core-shell microspheres-gelatin coating-3D printing titanium alloy porous scaffold. Reproduced with permission (Liu et al., 2022c).

By using additive manufacturing techniques, porous titanium scaffolds and their biomodification can be established, and the integration of which into bone can be significantly enhanced. On the surface of these titanium scaffolds, a hydrogel containing growth factors that promote osteogenesis and angiogenesis is coated. This composite system synergistically promotes the osteogenic differentiation of bone marrow mesenchymal stem cells (BMSCs) *in vitro* and accelerates the migration of human umbilical vein endothelial cells (HUVECs) as well as the formation of blood vessels *in vitro*, compared to a single growth factor. This composite system is capable of sustained releasing VEGF and BMP-9 in a sustained and coordinated manner to act on the localized bone defect area, thereby enhancing the integration of the bone-scaffold interface and improving the initial and long-term stability of the implant in the bone defect area (Che, 2023). This approach provides a new therapeutic avenue for bone defect management (Figure 9).

#### 3.5.3 Antibacterial properties

Traditional titanium implants lack antimicrobial properties, which may lead to post-implantation infections and patient discomfort (Alvand et al., 2017). Current clinical practice often involves 6–8 weeks of intravenous antibiotic therapy. However, this approach has several drawbacks, including difficulty in accurately managing antibiotic duration, low drug concentration at the target site, and the inability to achieve effective drug concentrations on the implant surface or within biofilms. Consequently, some patients require revision surgery, increasing their economic burden and that of society.

The antimicrobial mechanism of porous titanium implants involves both physical and chemical aspects. Physically, surface roughness and hydrophilicity can influence bacterial adhesion (Wu et al., 2011). Gasik et al. (2012) demonstrated that porous titanium with appropriate roughness and hydrophilicity significantly reduces bacterial adhesion compared to unmodified titanium surfaces. This also promotes cell proliferation and differentiation to some extent. Chemically, incorporating antibacterial metals such as copper or silver into titanium alloys can effectively inhibit bacterial growth and propagation (Xu et al., 2023).

Antibiotics remain the cornerstone for preventing and treating periprosthetic infections, despite extensive research into alternative antimicrobial methods such as metal ions and antimicrobial peptides (Kapadia et al., 2016). Antibiotic-loaded materials, like bone cements, are widely accepted for their effective antibacterial activity, with local delivery reducing systemic toxicity compared to intravenous administration (Spriano et al., 2018). Recent studies have explored topical antibiotic delivery through surface coatings, but additively manufactured porous materials offer superior drug-delivery potential (Orapiriyakul et al., 2018; Bakhshandeh et al., 2017).

Additive manufacturing enables the creation of titanium implants with specialized designs that enhance functionality. For instance, cavities within these implants can be filled with secondary therapeutic agents such as calcium phosphate or hydroxyapatite cement, forming lattice structures or external storage layers (Bezuidenhout et al., 2018). Parent et al. (2016) demonstrated enhanced efficacy against drug-resistant *S. aureus* by loading vancomycin onto porous hydroxyapatite surfaces. Additionally, broad-spectrum antimicrobial coatings have shown promise in combating diverse bacterial infections. Jianxin et al. (2019) combined levofloxacin, tinidazole, and methylprednisolone with a porous organic polymer to achieve broad-spectrum antimicrobial activity, including anti-inflammatory effects. Other antibiotics, such as etimicin (van Oosten et al., 2013), ceftriaxone (Kundu et al., 2011), and amoxicillin (Kazek-Kęsik et al., 2019), have also been incorporated into antimicrobial coatings.

### 3.6 Environmental response

**FIGURE 10:**
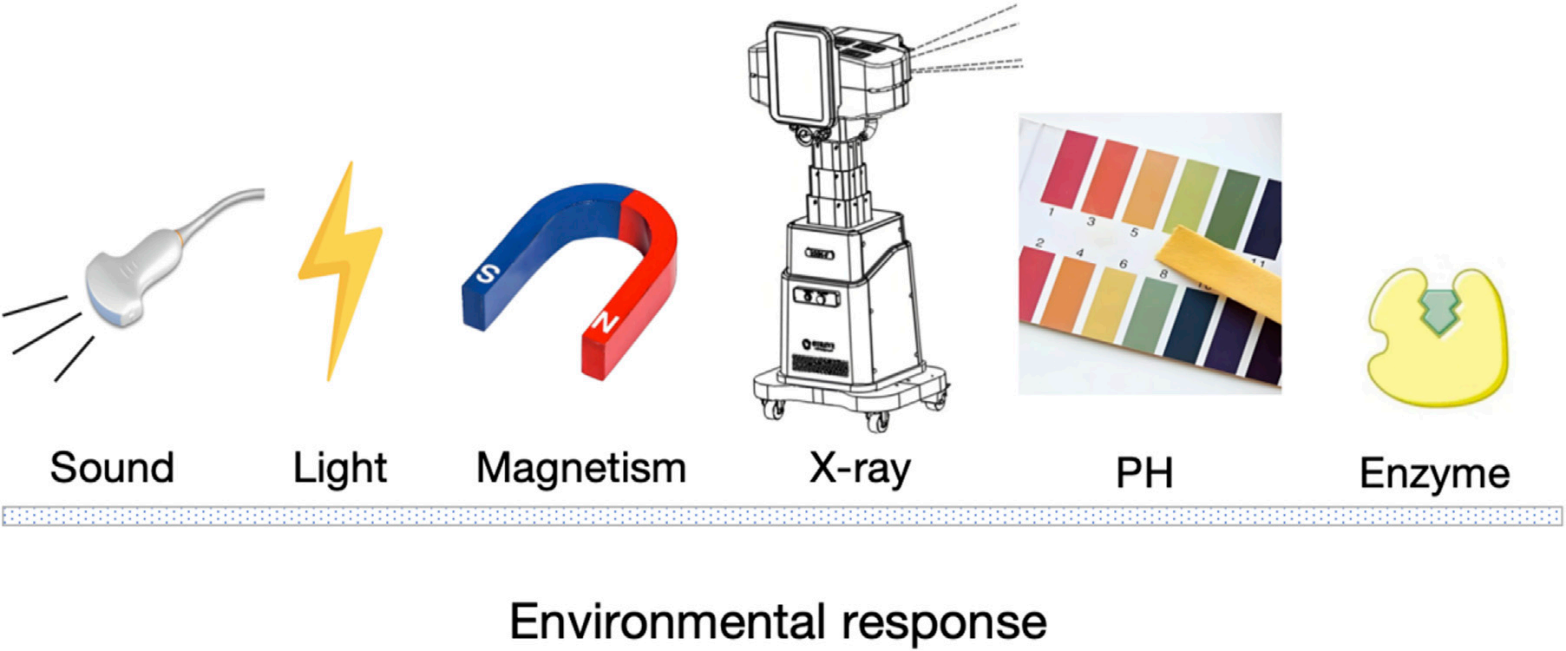
Environmentally responsive surfaces of porous titanium alloys.

While traditional materials have specific surface characteristics and structural morphology, biomaterials are evolving from traditional static to dynamic design (Tibbitt et al., 2015). Traditional orthopedic implants provide mechanical support and osseointegration functions, which can already be properly accomplished with the development of porous titanium alloy technology. At the same time, advances in material design, dynamic chemistry and nanotechnology allow us to introduce dynamic response properties into the surface modification of titanium alloys, utilizing acoustic, optical, electromagnetic as well as pH and enzyme (Zarubova et al., 2022), in conjunction with specific coatings to externally stimulate function (Bril et al., 2022). After much research and effort, a series of environmentally responsive surfaces have been successfully constructed on titanium alloy substrates. These surfaces have the ability to regulate cellular signaling, reduce the risk of infection by precisely controlling the release of drugs, and are important for revealing the subsequential biological processes inside cells (Figure 10).

#### 3.6.1 Ultrasound

Ultrasound, as a mechanical wave, is capable of directly modulating cell-material interactions and controlling drug release in a non-invasive manner. Low-intensity pulsed ultrasound has been approved by the U.S. Food and Drug Administration (FDA) for improving bone tissue reconstruction in fresh fracture healing and delayed fracture healing (Liang et al., 2022). Studies have shown that low-intensity pulsed ultrasound combined with micro-arc oxidation technology, where low-intensity ultrasound vibration affects the permeability of cell membranes and intracellular material movement, enhances the binding of MG63 cells to the surface, accelerates proliferation, and improves the expression of osteogenic differentiation (Chen et al., 2019b).

Ultrasound can modulate the rate of drug release, for instance, Aw and Losic (2013) reported micelles containing an insoluble drug (indomethacin) in response to ultrasound stimulation. Ultrasound interacts with an aqueous buffer and rigid titanium nanotube structures to generate cavitation and heat, which induces the release of the drug from the super-hydrophobic titanium nanotube arrays, and is even able to achieve 100% release in the desired time.

Ultrasound therapy can also be combined with thermotherapy, where ultrasound waves penetrate deeply into tissues and are spatially focused to a single discrete point. Red phosphorus-coated titanium surfaces (Ti-RP) exhibited a significant increase in acoustic-thermal activity under ultrasound excitation, related to the ultrasound-activated conversion of mechanical energy into phonons by electronic motion. The Ti-RP surfaces were functionalized with nanoporous silica nanoparticles containing thermoresponsive NO precursor particles coating for functionalization, the antimicrobial efficiency against multidrug-resistant *S. aureus* (MRSA) under ultrasonic excitation was more than 93.40%, which was effective against MRSA bone infection without side effects (Wang et al., 2020b).

#### 3.6.2 Light

Light as a non-invasive physical stimulus has demonstrated its potential for precise control at the microscopic level (Tian et al., 2022). Light is able to activate titanium oxide, which in turn modulates cellular behavior in both the temporal and spatial dimensions. Through this mechanism, we were able to utilize light to control drug release, enhance antimicrobial efficacy, and promote cell and tissue repair.

Titanium oxide possesses potential as a drug carrier but suffers from uncontrolled release. To address this challenge, researchers have adopted a series of innovative strategies. Song et al. (2009) achieved precise control of drug release by immobilizing APTES vitamin C monolayer linkers on TiO_2_ nanotubes and using ODPA to close the ends of the nanotubes. When these structures were exposed to UV light, the photocatalytic properties of TiO_2_ were able to break down the ODPA and the linkers, thereby controlling the release of the enzyme molecules. Given the limited penetration of UV light, the researchers further added noble metals capable of generating surface plasmon resonance to the TiO_2_ nanotubes, enabling the drug release process to take place under visible or infrared light. This technology not only regulates drug release, but can also be used directly to rapidly remove biofilms without causing tissue damage.

Despite the great potential for clinical applications, the single mode of photodynamic action has the disadvantages of short lifetime as well as limited diffusion distance (Deng et al., 2021), for this reason, researchers have combined photodynamic and photothermal properties to enhance the photoconversion efficiency and enhance the antimicrobial ability. Xu J. et al. (2016) prepared a red phosphorus-IR780-RGDC coating on the surface of a titanium alloy, with red phosphorus acting as a photothermite and IR780 as a photosensitizer, which can eliminate bacteria more efficiently when exposed to near infrared light irradiation, which can generate both heat and singlet oxygen, resulting in improved drug release efficiency as well as enhanced antimicrobial capacity.

#### 3.6.3 Electricity and magnetism

Electrical stimulation is a method of modulating cellular or tissue responses through external electrical signaling and involves a variety of behaviors and mechanisms, such as angiogenesis, mitosis, migration, and healing (Casella et al., 2021). It was shown that the proliferation and differentiation of osteoblasts were enhanced by biphasic electrical stimulation on the surface of titanium nanotubes (Ercan and Webster, 2010). A constant electric field was found to improve osteogenic differentiation of cloned rat MSCs even in the absence of bone-enhancing agents (Park et al., 2016). Although the behavior of cells stimulated by the application of electrical signals has been widely observed, the molecular mechanisms remain poorly understood (Ercan and Webster, 2010).

Mazare et al. (2019) prepared Arh2-reduced black TiO_2_ nanotube arrays, which significantly reduced the resistivity, and the enhanced conductivity of the TiO_2_ nanotube surface could trigger higher Ca^2+^ influx with minimal electric field stimulation, and increased of Ca^2+^ ions further regulated downstream signaling and improved cell proliferation.

Magnetic fields originate from static magnets or the flow of charge through source-based wires or electrical devices, which show high tissue penetration and minimal detrimental effects at low intensity magnetic fields (Jayasree et al., 2021). Magnetic fields can be utilized to trigger drug release using magnetic nanoparticles and stimulate cellular and tissue behaviors. Aw et al. (2012) prepared magnetically responsive delivery platforms on titanium nanotube arrays loaded with dopamine-modified iron oxide nanoparticles (MNPs) at the bottom and indomethacin-encapsulated polymer micelles at the top, achieving drug release controlled by an external magnetic field generated by the movement of electric magnetic fields. Fassina et al. (2008) constructed titanium alloy fiber mesh scaffolds and observed higher proliferation and matrix deposition of SAOS-2 cells under electromagnetic pulses, osteoblasts would be oriented along the magnetic field lines, and proliferation and expression of osteogenesis-related genes were upregulated.

#### 3.6.4 X-rays

X-rays, a form of ionizing radiation, are widely used in clinical diagnosis and therapy due to their high tissue penetration capacity and low autofluorescence background (Ying et al., 2022). X-rays are able to modulate osteoblast activity, trigger drug release, and assist in imaging of implant infections. Low-dose X-ray irradiation is beneficial for somatic osteoblast differentiation and mineralization, and also contributes to mineralization of fracture healing tissue *in vivo* (Karim and Judex, 2014). It has been shown that low-dose X-ray irradiation enhances bone growth towards the prosthetic surface, contributes to the stability of the prosthesis in the presence of wear particles, and inhibits the early development of aseptic loosening (She et al., 2016). Based on the response capability of TiO_2_, X-rays can be used to trigger drug release. Schmidt-Stein et al. (2009) used X-rays to induce the generation of electron-hole pairs from compact TiO_2_ layers and nanotube arrays to degrade organic compounds. Demonstrating the feasibility of X-ray activation for modulating potential drug release patterns.

X-ray excitation luminescence chemical imaging (XELCI) is a non-invasive technique used to detect and monitor bacterial infections on implant surfaces (Behbahani et al., 2022). The technique combines an X-ray scintillator layer and a pH-sensing layer to produce high-resolution images and optically detect the extent of infection through X-ray excitation. The ability of XELCI technology to image both acidic and basic pH regions around implants has shown potential applications for detecting local pH changes under acidotic conditions, including tumors, inflammation, and ischemia (Uzair et al., 2019), providing new strategy for implant infection treatment.

#### 3.6.5 pH

The ph response is achieved by pH-triggered protonation and deprotonation of chemical groups in the polymer cap of titanium surfaces (Lv et al., 2020). Chitosan, which is rich in amino groups, is one of the commonly used molecules for surface modification of titanium (Ning et al., 2019), and can be prepared to produce pH-sensitive coatings, which are capable of inhibiting bacterial growth by releasing more drugs, such as tobramycin, in acidic conditions (e.g., infected areas). This smart coating not only reduces bacterial adhesion, but also maintains the antimicrobial effect for a long time without harming normal cells (Zhou et al., 2018).

In addition to chitosan, polymethylmethacrylic acid (PMAA) has also been used to prepare pH-sensitive materials. Such material swells at neutral pH and shrinks under acidic conditions, thereby controlling drug release. Chen et al. (2020b) used PMAA to encapsulate antimicrobial peptides (AMPs) to prolong drug release under physiological conditions and rapidly release AMPs to kill bacteria under infected conditions. In addition, some pH-sensitive chemical bonds such as metal-ligand bonds and Schiff bases have also been used in the preparation of coatings (Wang et al., 2017c; Tao et al., 2019), which can be fractured in acidic environments to release the drug and effectively eliminate bacteria.

The pH-responsive mode can also be combined with other therapeutic approaches, such as catalytic therapy and gas therapy, for the treatment of infected joints. This strategy works by depositing special nanoparticles on titanium surfaces that trigger a chemical reaction in a bacterial-induced acidic environment, generating reactive oxygen species (ROS) and oxygen capable of killing bacteria while alleviating the hypoxic environment of the infected area (Zhang et al., 2022b).

#### 3.6.6 Enzymes

Enzyme-responsive modes typically utilize enzymes to hydrolyze coatings as a way to trigger drug release or activate specific cellular behaviors. Hyaluronidase, for example, is commonly used to cleave coatings containing hyaluronic acid in order to modulate drug release kinetics. Yuan et al. (2018) loaded vancomycin (Van) into TiO_2_ nanotubes, and then sealed the titanium nanotubes using alternating layers of 3,4-dihydroxycinnamic acid-modified chitosan (Chi-c) and dopamine-modified hyaluronic acid (HA-c). In the presence of hyaluronidase or *S*. *aureus*, the degradation of the multilayered membrane controlled the titanium nanotubes to release more than 50% of Van within 24 h with an antimicrobial rate higher than 80%. At the same time, this hybridized surface significantly improved the initial adhesion of rat cranial osteoblasts and significantly cleared the surrounding medulla of infection. In some cases, hyaluronic acid can be further combined with antimicrobial agents. Yu et al. (2020) used sodium hyaluronate-laurate coupling or hyaluronic acid-gentamic acid coupling to cap BMP-2 or desferrioxamine-loaded titanium nanotubes, respectively. The designed and modified surfaces exhibited not only antifouling and antimicrobial properties, but also improved osteogenic differentiation.

In addition, micrococcal nuclease and serine protease-like protease (SplB) secreted by *S. aureus* have been used to trigger the surface reaction. Ghimire et al. (2019) combined vancomycin with a polyethylene glycol dimethacrylate hydrogel coating on a titanium alloy surface with an oligonucleotide junction that was susceptible to *S. aureus* micrococcal nuclease (MN). The MN-triggered release of the Vancaused prompt eradication of *S. aureus* around the implant surface and effectively tethered vancomycin doses prevented *S. aureus* infections in mouse femoral tubes more than 200-fold lower than the amount of prophylactic antibiotics used in bone cement.

In addition to bacterial-associated enzymes, osteoblast-associated enzymes are also used to modify surface reactions. A coating consisting of acetylglucosan (acBSP) and alendronate (ALN) was constructed on the surface of a titanium alloy, and *in vitro* assays showed that acBSP activated macrophages to express osteoclastogenic cytokines containing oncostatin M, which is a member in the pro-inflammatory cytokines of interleukin-6 family of, and suppresses pro-inflammatory macrophages and directly induces osteogenesis. *In vivo* results in an osteoporotic rat model showed that this bioresponsive coating significantly improved bone-implant contact rates, which provides new ideas for immunomodulatory surface modifications (Wang et al., 2024b).

## 4 Current challenges and future prospects

Porous titanium alloy implants play an important role in modern orthopedic medicine, especially in surgeries such as fracture fixation, spinal fusion, joint replacement, and bone tumor defect repair. However, despite their clinical success, these implants still face some challenges and have set new requirements for future development.

With the increasing demand for personalized medical care, traditional implant manufacturing methods cannot meet the needs of patients with specific anatomical and skeletal characteristics. Although additive manufacturing technology offers the possibility of manufacturing personalized implants, most orthopedic surgeons are unfamiliar with the operation methods of related software and equipment, which limits the popularization and development of this technology. At the same time, due to the diversity and rapid development of 3D printing technology, there are currently various types of 3D printing equipment, methods and materials, thus there are seldom unified clinical application specifications and standards, which limits the further development of the technology.

In the future, the combination of 4D printing and Artificial intelligence technology will make it possible to design more personalized and intelligent orthopedic implants that can self-adjust and optimize according to the specific conditions of the patient. The development of new bioactive coatings and drug release systems to promote osseointegration, angiogenesis, and infection control will be the focus of future research. As the technology develops, an international standard evaluation system needs to be established to ensure the quality and safety of customized implants while also addressing related ethical issues.

At present, most of the additive manufacturing porous titanium alloy technology is still limited to animal experiments and is still some way from clinical application and translation. There is still considerable gap for research in the field of orthopedics in the future. The future of additive manufacturing porous titanium alloy implants in orthopedics will focus more on personalized, intelligent and precision medicine, while interdisciplinary cooperation is needed to integrate material science, bioengineering, information technology and clinical needs to achieve safer and more effective treatment options, stimuli-and environment-responsive device, and jointly promote the progress and development of orthopedic medicine.
